# Model-driven approach for the production of butyrate from CO_2_/H_2_ by a novel co-culture of *C. autoethanogenum* and *C. beijerinckii*

**DOI:** 10.3389/fmicb.2022.1064013

**Published:** 2022-12-22

**Authors:** Sara Benito-Vaquerizo, Niels Nouse, Peter J. Schaap, Jeroen Hugenholtz, Stanley Brul, Ana M. López-Contreras, Vitor A. P. Martins dos Santos, Maria Suarez-Diez

**Affiliations:** ^1^Laboratory of Systems and Synthetic Biology, Wageningen University and Research, Wageningen, Netherlands; ^2^Molecular Biology and Microbial Food Safety, University of Amsterdam, Amsterdam, Netherlands; ^3^UNLOCK Large Scale Infrastructure for Microbial Communities, Wageningen University and Research and Delft University of Technology, Wageningen, Netherlands; ^4^Wageningen Food and Biobased Research, Wageningen University and Research, Wageningen, Netherlands; ^5^Bioprocess Engineering, Wageningen University and Research, Wageningen, Netherlands; ^6^LifeGlimmer GmbH, Berlin, Germany

**Keywords:** microbial communities, *Clostridium autoethanogenum*, *Clostridium beijerinckii*, constraint-based metabolic modeling, CO_2_, H_2_, lactate, butyrate

## Abstract

One-carbon (C1) compounds are promising feedstocks for the sustainable production of commodity chemicals. CO_2_ is a particularly advantageous C1-feedstock since it is an unwanted industrial off-gas that can be converted into valuable products while reducing its atmospheric levels. Acetogens are microorganisms that can grow on CO_2_/H_2_ gas mixtures and syngas converting these substrates into ethanol and acetate. Co-cultivation of acetogens with other microbial species that can further process such products, can expand the variety of products to, for example, medium chain fatty acids (MCFA) and longer chain alcohols. Solventogens are microorganisms known to produce MCFA and alcohols *via* the acetone-butanol-ethanol (ABE) fermentation in which acetate is a key metabolite. Thus, co-cultivation of an acetogen and a solventogen in a consortium provides a potential platform to produce valuable chemicals from CO_2_. In this study, metabolic modeling was implemented to design a new co-culture of an acetogen and a solventogen to produce butyrate from CO_2_/H_2_ mixtures. The model-driven approach suggested the ability of the studied solventogenic species to grow on lactate/glycerol with acetate as co-substrate. This ability was confirmed experimentally by cultivation of *Clostridium beijerinckii* on these substrates in batch serum bottles and subsequently in pH-controlled bioreactors. Community modeling also suggested that a novel microbial consortium consisting of the acetogen *Clostridium autoethanogenum*, and the solventogen *C. beijerinckii* would be feasible and stable. On the basis of this prediction, a co-culture was experimentally established. *C. autoethanogenum* grew on CO_2_/H_2_ producing acetate and traces of ethanol. Acetate was in turn, consumed by *C. beijerinckii* together with lactate, producing butyrate. These results show that community modeling of metabolism is a valuable tool to guide the design of microbial consortia for the tailored production of chemicals from renewable resources.

## 1. Introduction

The energy crisis and the effects of climate change have emphasized the need to accelerate the transition toward a circular bio-based economy (Gottinger et al., 2020). Current circular approaches focus on the application of microbial conversion to convert low-value carbon feedstocks, such as biomass waste streams, into commodity chemicals (Casau et al., 2022). Recalcitrant lignocellulosic biomass can be pretreated and hydrolyzed into sugars (Loow et al., 2016) or gasified to produce synthesis gas (syngas), a one-carbon (C1) feedstock consisting of a mixture of CO, CO_2_ and H_2_ (Richardson et al., 2015). In addition, C1-rich industrial waste gases from steel and thermal power plants can be directly used as microbial feedstocks (Hwang et al., 2020). In this regard, CO_2_ is an advantageous one-carbon feedstock (C1) since it can be obtained from natural and industrial sources, and can be converted into valuable products reducing the release of contaminant gases to the environment.

Acetogens are strict anaerobes that can grow on CO_2_/H_2_ and syngas as their sole carbon source using the Wood-Ljungdahl metabolic pathway (Ragsdale and Pierce, 2008; Bertsch and Müller, 2015). Acetogenic fermentation of C1 gases leads mainly to the production of acetate and ethanol, as well as, 2,3-butanediol or lactate (Köpke et al., 2014; Valgepea et al., 2018), and the production of ethanol has been commercialized recently (Marcellin et al., 2016; Bae et al., 2022). Acetogens that grow autotrophically on these slightly soluble gases are energy limited and produce a constrained product spectrum and low product titers. This can be overcome by exploring alternative strategies, such as mixotrophic growth or co-cultivation (Lee et al., 2022).

Solventogenic Clostridia have been widely applied to ferment sugars into mixtures of the solvents acetone, butanol, and ethanol (ABE) (Tracy et al., 2012) or in some cases isopropanol, butanol, ethanol (IBE) (Yang et al., 2016). These fermentations consist of an acidogenic phase followed by a solventogenic phase. During acidogenesis, solventogens produce carboxylic acids (mainly acetate and butyrate), and CO_2_ and H_2_. Accumulation of carboxylic acids and the concomitant lowering of the pH trigger solventogenesis during which solventogens reduce the carboxylic acids into solvents (Liao et al., 2015).

Cross-feeding strategies have been used to establish synthetic microbial communities that produce a wider product range (Diender et al., 2016, 2019; Du et al., 2020; Moreira et al., 2021). Therefore, co-cultivation of an acetogen and a solventogen has the potential to overcome the drawbacks associated to acetogens by increasing the product spectrum. Charubin and Papoutsakis (2019) recently established a co-culture of the solventogen *Clostridium acetobutylicum* and the acetogen *Clostridium ljungdahlii*. In this setup, glucose was metabolized by *C. acetobutylicum* to butanol, ethanol, acetone, acetoin, CO_2_, and H_2_. Subsequently, CO_2_ and H_2_ were fixed, and acetone and acetoin were reduced to isopropanol and 2,3-butanediol, respectively, by *C. ljungdahlii*.

Acetate is one of the most abundant products in acetogens (Bengelsdorf et al., 2018), and while solventogenic strains cannot grow on acetate as sole carbon source, they can reassimilate acetate and convert it into carboxylic acids such as butyrate, or solvents when glucose is used as co-substrate (Monot et al., 1982; Kuit et al., 2012; Diallo et al., 2021). Therefore, in a co-culture of acetogens/solventogens on CO_2_/H_2_, butyrate could be produced as main product. Butyrate is a valuable product as it is used in many commercial applications, as a solvent, cosmetic, food, animal feed, or as a precursor of pharmaceuticals (Dwidar et al., 2012; Brändle et al., 2016).

In this study, we followed a model-driven approach to find an alternative route for production of butyrate. We produced butyrate from CO_2_ using a co-culture of two strains, one acetogen producing acetate from CO_2_/H_2_, and one solventogenic strain that co-metabolized acetate with an alternative carbon source into butyrate. To select the solventogenic strain, we systematically assessed growth on several carbon sources using the genome-scale metabolic models (GEMs) of *C. acetobutylicum* and *Clostridium beijerinckii*. The most promising carbon sources were experimentally tested and validated. On the basis thereof, we constructed a community model of *Clostridium autoethanogenum* and *C. beijerinckii*, and qualitatively assessed the fermentation of CO_2_/H_2_ and the new carbon source through scenario simulations. Model predictions guided the experimental work and led to the successful establishment of this new co-culture.

## 2. Materials and methods

### 2.1. GEM availability and curation

The GEM of *C. autoethanogenum* DSM 10061, iCLAU786 (Valgepea et al., 2017) was downloaded in sbml and table format and used without modification.

The GEM of *C. acetobutylicum* ATCC 824, iCac802 (Dash et al., 2014) was downloaded in sbml and table format, and modified as follows: Two reactions were defined as reversible: ATP:3-phospho-d-glycerate 1-phosphotransferase (with model identifier R0239), and Hydrogenase (R1563). Formate dehydrogenase (R1562) was removed since it was not found in the genome of *C. acetobutylicum*. Three new reactions were added in the model: Pyruvate transport (pyrt), Pyruvate exchange (EX_PYR_e), and glycerol kinase (R0426), the latter reaction was found to be present in the genome of *C. acetobutylicum* (EC 2.7.1.30; locus tag: CAC1321). Finally, we replaced the ethanol transport reaction (R1708), expressed as a proton (H^+^) symport reaction, by a diffusion transport reaction. The modified version of the model (iCac803) is available at: https://gitlab.com/wurssb/Modelling/coculture_cacb.

The GEM of *C. beijerinckii* NCIMB 8052, iCM925 (Milne et al., 2011) was downloaded in sbml and table format and modified as follows: Ferredoxin-NAD^+^ reductase (FDXNRx) and ferredoxin-NADP^+^ reductase (FDXNRy) reactions were removed, and Na^+^-translocating ferredoxin:NAD^+^ oxidoreductase (Rnf) complex and electron-bifurcating, ferredoxin-dependent transhydrogenase (Nfn) complex, were added to the model. Transport of hydrogen reaction (Habc) was replaced by the ATPase reaction (ATPase). The EC number and genes of (*S*)-3-Hydroxybutanoyl-CoA:NADP^+^ oxidoreductase reaction (HACD1y) were modified and the EC numbers of butyryl-CoA dehydrogenase (ACOAD1) and 3-hydroxybutyryl-CoA dehydrogenase (HACD1x) as well. Finally, the reduced ferredoxin:dinitrogen oxidoreductase (ATP-hydrolyzing) reaction (DNOR) was stoichiometrically balanced. The updated version of the model (iCM943) is available in the git repository.

### 2.2. *In-silico* carbon source screening in *C. acetobutylicum* and *C. beijerinckii*

We systematically assessed growth capabilities on the updated versions of the GEMs of *C. acetobutylicum* and *C. beijerinckii* on a wide range of carbon sources. Model simulations were done using COBRApy, version 0.15.4 (Ebrahim et al., 2013), and Python 3.9. Growth capabilities were assessed using Flux Balance Analysis (FBA). The biomass synthesis reaction (termed “Biomass” or “biomass” in the respective *C. acetobutylicum* and *C. beijerinckii* models), was defined as the objective function for maximization. Growth was considered when the growth rate was higher than 0.0001 h^-1^. For each assessed carbon source, the maximum uptake (corresponding to minus the lower bound of the associated exchange reaction denoted “EX_xx”) was constrained to 20 mmol g_DW_^-1^ h^-1^, and the minimum uptake (corresponding to minus the upper bound of the associated exchange reaction) was constrained to 0.1 mmol g_DW_^-1^ h^-1^. In addition, uptake of small metabolites and ions was allowed by setting the lower bound of the corresponding exchange reaction to −1,000 as described in the corresponding script in the git repository.

### 2.3. Co-culture GEM reconstruction

A compartmentalized co-culture model of *C. autoethanogenum* and *C. beijerinckii* was obtained by combining single species models iCLAU786 (Valgepea et al., 2017) and iCM943 (Milne et al., 2011), following a previous approach (Benito-Vaquerizo et al., 2020). In this approach, each species is considered a single compartment. The compartment associated with *C. autoethanogenum* was defined as “cytosol_ca” and the compartment associated with *C. beijerinckii* was defined as “cytosol.” Intracellular metabolites were assigned to their respective compartment and the flag “_ca” was added to the identifier of metabolites belonging to “cytosol_ca” to distinguish them from the *C. beijerinckii* metabolites. In addition, the combined model included an extracellular compartment, defined as “extracellular,” common to both species. Metabolites in this compartment are either secreted, metabolized, or exchanged by both species, and separated from metabolites present in the cellular compartments by adding the “_e” flag to the identifier. All extracellular metabolites follow the same naming system (namespace) for both species. Therefore, the same namespace was applied to metabolites secreted by both species. All metabolites present in both intracellular compartments and the extracellular compartment can be exchanged between species if favored by the directionality. Interchanged metabolites are assumed to be first transported into the extracellular compartment, before taken up by the other species using the corresponding exchange reaction. The co-culture GEM contains one biomass synthesis reaction per species, termed “biomass_auto” and “biomass_beije” for *C. autoethanogenum* and *C. beijerinckii*, respectively. Additionally, the model contains a community biomass synthesis reaction (“Community_biomass”), which incorporates the biomass of *C. autoethanogenum* and *C. beijerinckii* in the form of metabolite “biomass_ca” and metabolite “biomass,” respectively. The combined model incorporates a transport reaction of butyrate (“BUTex_au”) from the extracellular compartment to the intracellular compartment of *C. autoethanogenum*, and the reaction to produce butyraldehyde from butyrate (“buttobuta”) in *C. autoethanogenum*. In addition, we incorporated the transport reaction of acetone (“ACETONE_ca”) from the extracellular compartment to the intracellular compartment of *C. autoethanogenum*; an alcohol dehydrogenase to convert acetone into isopropanol (“ISOBIO”); a transport reaction of isopropanol from the intracellular compartment of *C. autoethanogenum* to the extracellular compartment (“ISOPRO_ca”), and an exchange reaction of isopropanol (“EX_IPRO_e”).

The final three-compartment co-culture model was translated into SBML level 3 version 1 (see git repository).

### 2.4. Co-culture modeling framework

Co-culture model simulations were carried out using a previously described modeling framework (Benito-Vaquerizo et al., 2020), similar to SteadyCom (Chan et al., 2017), and based on Community FBA (cFBA) (Khandelwal et al., 2013). Specific fluxes (mmol g_DW_^−1^ h^−1^) were substituted by environmental fluxes (mmol l^−1^ h^−1^), and thus, the biomass synthesis reaction of each species was changed accordingly accounting for the growth rate and biomass of each species (g_DW_ l^−1^ h^−1^). The relative contribution of each species to the community biomass was calculated from the total biomass of the community and the species ratio.

### 2.5. Co-culture model simulations

In this study, we simulated hypothetical scenarios varying biomass species ratios, growth rates, and substrates environmental fluxes to explore the feasible solution space of the co-culture. We selected a community biomass of 0.22 g_DW_ l^-1^ based on the average value measured for a similar co-culture of *C. autoethanogenum* and *Clostridium kluyveri* on syngas (Diender et al., 2019). The biomass of each species was calculated based on the indicated species ratio and the community biomass. The biomass of each species was multiplied by the indicated growth rate, and the value was used to constrain the flux through the biomass synthesis reaction of each species. We assessed conditions with *C. autoethanogenum*–*C. beijerinckii* ratios ranging from 0.1–0.9 to 0.9–0.1, and growth rates from 0.005 to 0.1 h^-1^. For this exploratory analysis, we assumed equal growth rates for each species and steady-state. For each condition, we fixed the uptake rate of CO_2_ and H_2_ to 5 mmol l^-1^ h^-1^ or to 2.5 mmol l^-1^ h^-1^, covering values found in literature for a similar co-culture (Diender et al., 2019). The maximum lactate uptake rate was constrained to 2.5 or 5 mmol l^-1^ h^-1^ and a minimum uptake rate of 0.1 mmol l^-1^ h^-1^ was imposed. We defined the community biomass reaction (“Community_biomass”) as the objective function and we performed FBA to assess the feasibility of each condition. For a selected number of feasible conditions, the solution space and the set of fluxes compatible with the measured constraints were sampled using the *sample* function in the flux_analysis submodule of COBRApy. Presented results show the average and standard deviation based on 5,000 iterations generated at each condition (git repository).

### 2.6. Bacterial strains

The laboratory strains *C. beijerinckii* NCIMB 8052 and *C. acetobutylicum* ATCC 824 were stored as spore suspensions in 20% glycerol at −20°C. Spores of *C. beijerinckii* and *C. acetobutylicum* were heat-activated for 1 min at 95°C and 10 min at 70°C, respectively, before inoculation. *C. autoethanogenum* DSM 10061 was kindly provided by Professor Diana Z. Sousa from the Laboratory of Microbiology, Wageningen University and Research, Wageningen, the Netherlands, and was stored as vegetative cells suspended in 25% glycerol buffered with phosphate and reduced with Ti(III)citrate under anoxic conditions at −80°C.

### 2.7. Experimental carbon source screening of *C. acetobutylicum* and *C. beijerinckii*

Cultures were prepared in serum bottles containing CM2 medium consisting of the following components: 2.5 g l^-1^ yeast extract (Duchefa Biochemie), 1.0 g l^-1^ KH_2_PO_4_ (Fischer Scientific), 0.61 g l^-1^ K_2_HPO_4_ (Sigma-Aldrich), 1 g l^-1^ MgSO_4_ · 7H_2_O (Roth), 2.9 g l^-1^ ammonium acetate (USB), 0.10 g l^-1^ 4-aminobenzoic acid (Duchefa Biochemie), 6.6 mg l^-1^ Fe(II)SO_4_ · 7H_2_O (Sigma-Aldrich), and 0.5 mg l^-1^ Na-resazurin (Sigma-Aldrich). Acetic acid (Sigma-Aldrich), l-lactic acid (~90%, Merck), ethanol (Merck) and glycerol (Sigma-Aldrich) were added to final concentrations of 40 mM. pH was set to pH 6.1–6.2 with KOH and/or HCl. Media were made anoxic with N_2_(g) and autoclaved. d-Glucose was made anoxic and autoclaved separately and added to a final concentration of 40 mM. Media were inoculated with 4% (v/v) culture made from heat-activated spore suspension grown overnight at 37°C in CM2 medium supplemented with 40 g l^-1^ d-glucose (Duchefa Biochemie). Cultures were incubated at 37°C and sampled at t_0_ and after 4 d. Cell density was measured as optical density at 600 nm (OD_600_), extracellular metabolites were analyzed with high-performance liquid chromatography (HPLC) as described in Section 2.9, and pH was measured. Acetate co-assimilation was determined by calculating the fraction of the total converted carbon coming from consumed acetate.

### 2.8. Cultivation experiments in pH-controlled bioreactors

pH-controlled bioreactor experiments were performed in a working volume of 2 l in Infors HT Labfors 5 bioreactors (Infors HT, Switzerland). The stirrer, set at 150 rpm, consisted of at equidistance from top to bottom a pitch-blade and two Rushton impellers. Temperature was controlled at 37°C and pH at pH 5.5 ± 0.1 using 3 M KOH and 2 M H_3_PO_4_. Foaming was controlled with Antifoam 204 (Sigma-Aldrich). 2.9 g l^-1^ ammonium acetate in the CM2 medium was replaced by 2.5 g l^-1^ ammonium sulfate (Merck). 0.75 g l^-1^ anoxic and sterilized l-cysteine HCl · H_2_O (Merck) was added after autoclaving.

In pH-controlled batch fermentations of *C. beijerinckii* growing on different concentrations of acetate and lactate, the adapted CM2 medium was supplemented with acetic acid and l-lactic acid prior to autoclaving. 10 ml min^-1^ N_2_(g) was flushed across the head space to keep anoxic conditions. Reactors were inoculated with 1% (v/v) *C. beijerinckii* culture growing overnight at 37°C in CM2 medium supplemented with 20 g l^-1^ d-glucose and 0.75 g l^-1^ l-cysteine HCl.H_2_O. Cultures were sampled at regular time intervals for analysis of cell density, extracellular metabolites, and morphology with phase-contrast microscopy. The overall stoichiometry was calculated by scaling the difference of the concentrations of the main extracellular metabolites between t_0_ and t_end_ to the difference in the measured lactate concentration.

In pH-controlled fed-batch co-cultivation experiments of *C. autoethanogenum* and *C. beijerinckii*, reactors were equipped with sinter spargers to flush 40 ml min^-1^ H_2_(g) and 10 ml min^-1^ CO_2_(g) through the medium. At t_0_, reactors were inoculated with <1% (v/v) *C. autoethanogenum* culture growing at 37°C in CM2 medium supplemented with 10 g l^-1^ d-fructose (VWR Chemicals) and 0.75 g l^-1^ l-cysteine HCl.H_2_O. After establishment of growth and acetate production by *C. autoethanogenum*, reactors were inoculated with <1% (v/v) *C. beijerinckii* culture growing overnight at 37^o^C in CM2 medium supplemented with 20 g l^-1^ d-glucose and 0.75 g l^-1^ l-cysteine HCl.H_2_O. Furthermore, the continuous l-lactic acid feed was started. Cultures were sampled at regular time intervals for analysis of cell density, extracellular metabolites, and morphology with phase-contrast microscopy. Theoretical acetate production from CO_2_ was calculated for each time point after the start of the l-lactic acid feed as follows: The amount of lactate converted was calculated from the difference in the amount of lactate fed and calculated amount of lactate remaining in the reactor. The conversion of lactate *via* pyruvate yields the intermediate metabolite acetyl-CoA and CO_2_ in a 1:1 ratio. The fraction of the amount of carbon from lactate available for the formation of products was subtracted from the amount of carbon present in the produced (iso)butyrate to obtain a theoretical amount of carbon coming from a difference source than lactate, i.e., from converted acetate. This theoretical amount of converted acetate was added to the calculated amount of acetate in the reactor to get a theoretical amount of acetate produced from CO_2_. Subsequently, the stoichiometry for the production of (iso)butyrate from lactate and acetate was calculated by scaling the difference of (iso)butyrate produced, theoretical acetate converted, and lactate converted between t_0_ and the selected time points to lactate converted.

### 2.9. Analysis of extracellular metabolites

Concentrations of acetate, acetone, butanol, (iso)butyrate, ethanol, fructose, glycerol, glucose, and lactate were analyzed with HPLC. Supernatant was mixed with an equal volume of 1 M H_2_SO_4_ with 30 mM 4-methylpentanoic or 100 mM pentanoic acid as internal standard. This was filtered through a 0.2 μm regenerated cellulose filter followed by analysis on a Waters HPLC system with a Shodex KC-811 column at 65°C, 1 ml/min 3 mM H_2_SO_4_ mobile phase, and a refractive index and UV detector.

## 3. Results

**Figure 1:**
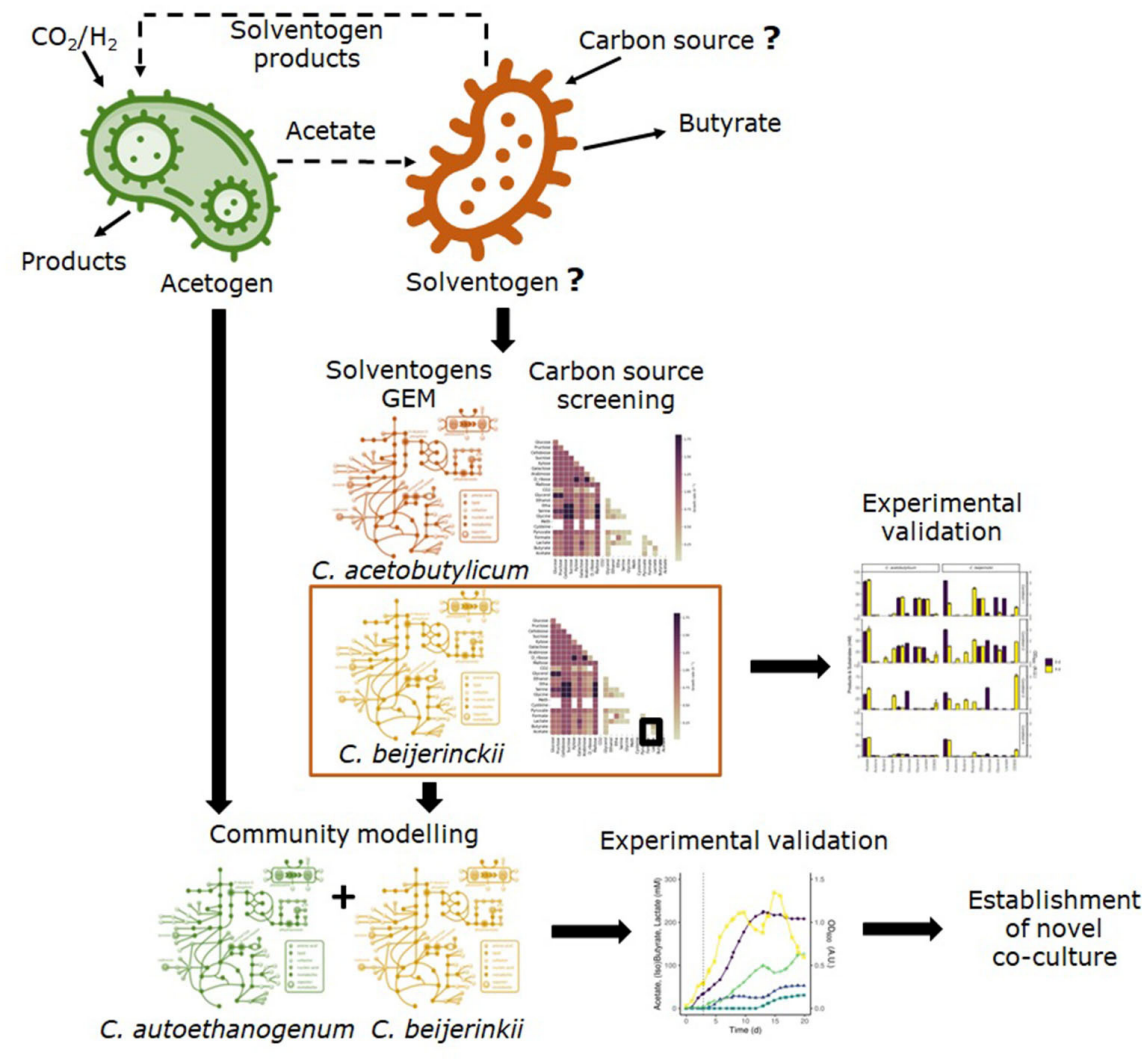
Overview of the followed methodology to establish a co-culture of an acetogen and a solventogen to produce butyrate from CO_2_/H_2_. We used genome-scale metabolic models of two solventogens to assess growth on several carbon sources and to find the alternative carbon source that sustained growth on the solventogen with acetate. The predicted carbon sources were experimentally validated and growth was confirmed in one of the solventogens. After that, we assessed the feasibility of the co-culture of the acetogen *C. autoethanogenum* and the selected solventogen using community modeling, and finally, the co-culture was experimentally established.

Figure 1 shows the steps for the model-driven approach followed to establish a novel co-culture of *C. autoethanogenum* and *C. beijerinckii* for the production of butyrate from CO_2_/H_2_. GEMs of solventogens were used to evaluate candidate species and possible carbon sources. After the experimental validation of the model predictions, the co-culture was successfully established.

### 3.1. Updated GEMs of *C. autoethanogenum, C. acetobutylicum*, and *C. beijerinckii*

The GEM of *C. autoethanogenum*, iCLAU786 was used as the original version (Valgepea et al., 2017) with no modification. The updated version of the GEM of *C. acetobutylicum*, iCac803 (Dash et al., 2014), had 1,254 reactions, 1,465 reactions, and 803 genes, and the GEM of *C. beijerinckii*, iCM943 (Milne et al., 2011), had 881 metabolites, 941 reactions, and 943 genes.

Regarding the GEM of *C. beijerinckii*, we included the Na^+^-translocating ferredoxin:NAD^+^ oxidoreductase (Rnf) complex (EC 7.2.1.2) in the model of *C. beijerinckii*. Rnf is formed by the following gene cluster: rnfC, rnfD, rnfG, rnfE, rnfA, rnfB, (locus_tag: Cbei_2449, Cbei_2450, Cbei_2451, Cbei_2452, Cbei_2453, and Cbei_2454, respectively). Additionally, we have identified an electron-bifurcating, ferredoxin-dependent transhydrogenase (Nfn) complex that catalyzes NADH-dependent reduced Ferredoxin:NADP^+^ oxidoreductase activity in *C. beijerinckii*. The Nfn complex has two subunits: NAD(P)-binding subunit, and a glutamate synthase subunit. These two subunits showed 56–79% identity with the Nfn subunits of *C. kluyveri, C. autoethanogenum, C. difficile* (Kremp et al., 2020), and of the recently annotated *Anaerotignum neopropionicum* (Benito-Vaquerizo et al., 2020), forming two possible complexes: (Cbei_2182 and Cbei_2183) or (Cbei_0661 and Cbei_0662). To our knowledge, this is the first time the Nfn complex is reported in *C. beijerinckii* NCIMB 8052. Ferredoxin NAD^+^ reductase (EC 1.18.1.3) is not found in the genome of *C. beijerinckii*, and the ferredoxin NADP^+^ reductase (EC 1.18.1.2) showed lower percentage identity to the FNR of *C. acetobutylicum*, and thus, we hypothesized that the former FNR corresponds to the NADP-binding subunit of the Nfn complex.

### 3.2. *In-silico* carbon source screening of *C. acetobutylicum* and *C. beijerinckii*

**Figure 2:**
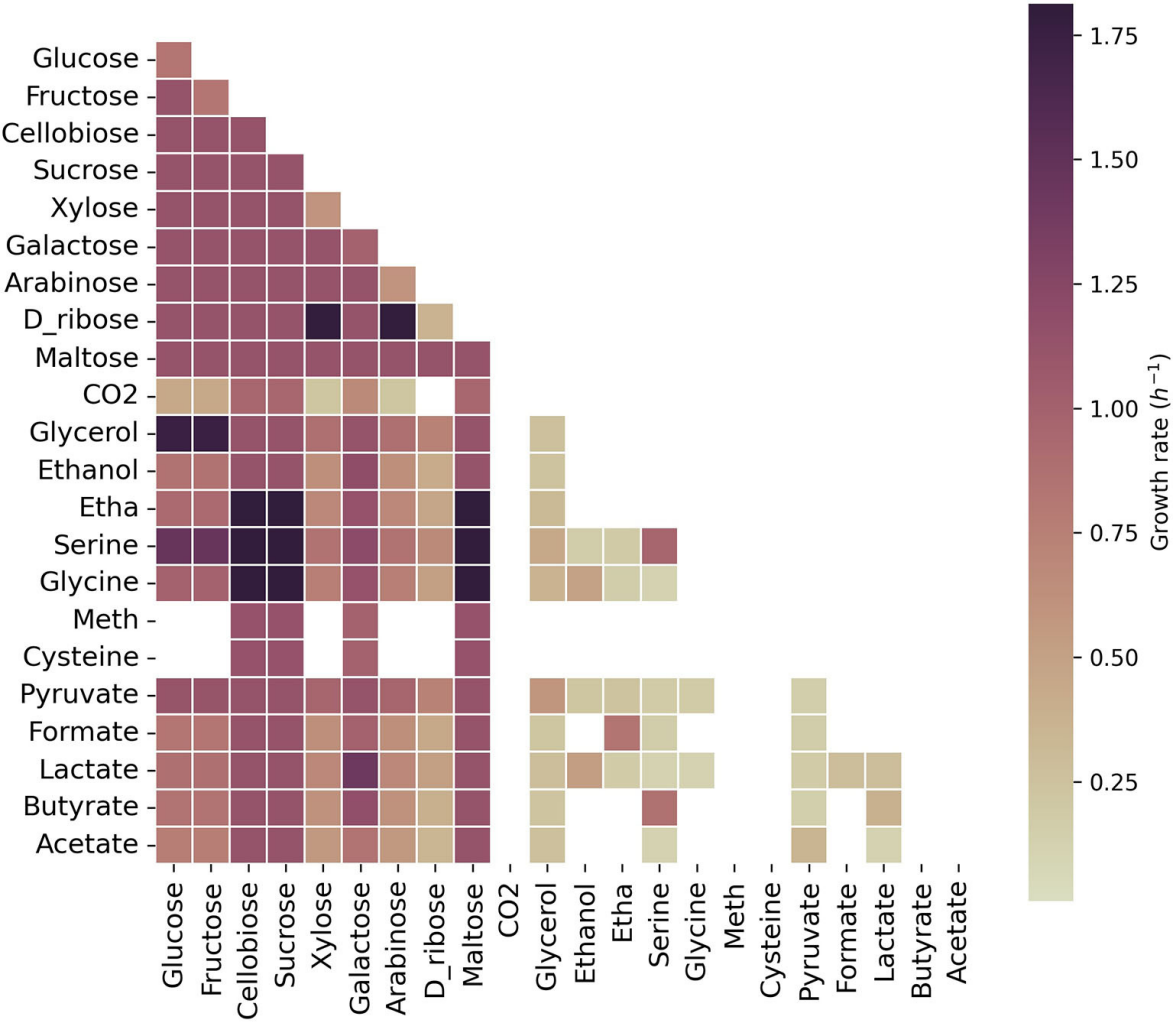
Growth capabilities predicted for *C. acetobutylicum*. The color scale shows growth rates in h^-1^. Squares on the diagonal correspond to single carbon sources. Squares below the diagonal correspond to the combination of the carbon source presented on the x-axis with the carbon source presented on the y-axis. Non-colored squares show that no growth was predicted for the specified carbon source(s). Meth, methionine; Etha, ethanolamine. The maximum uptake rate for each carbon source was set to 20 mmol g_DW_^-1^ h^-1^ and the minimum to 0.1 mmol g_DW_^-1^ h^-1^.

We explored growth on 25 carbon sources individually and pairs of these carbon sources with one another in the model of *C. acetobutylicum* (Figure 2).

Growth was predicted on sugars, glycerol, lactate, serine, and pyruvate as single carbon sources, reaching the highest growth rates on cellobiose, sucrose and maltose. As expected, acetate did not sustain growth as the sole carbon source and neither was sustained on acetone, succinate, acetoin (not shown here). Pairwise combinations of most carbon sources that led to growth as single carbon source, also led to growth in combination with an alternative carbon source. However, l-methione, l-cysteine and CO_2_ did not show growth in combination with carbon sources that sustained growth alone since the model was forced to uptake a minimum amount of each carbon source, leading in some cases, to infeasible solutions. The highest growth rates were obtained with cellobiose, sucrose or maltose in combination with serine, glycine or ethanolamine; the combination of glucose or fructose with glycerol, and xylose, or arabinose with ribose. Interestingly, acetate, in combination with lactate or glycerol, could sustain growth in *C. acetobutylicum*, as previously described for other solventogens (Diez-Gonzalez et al., 1995; Kumar et al., 2022).

**Figure 3:**
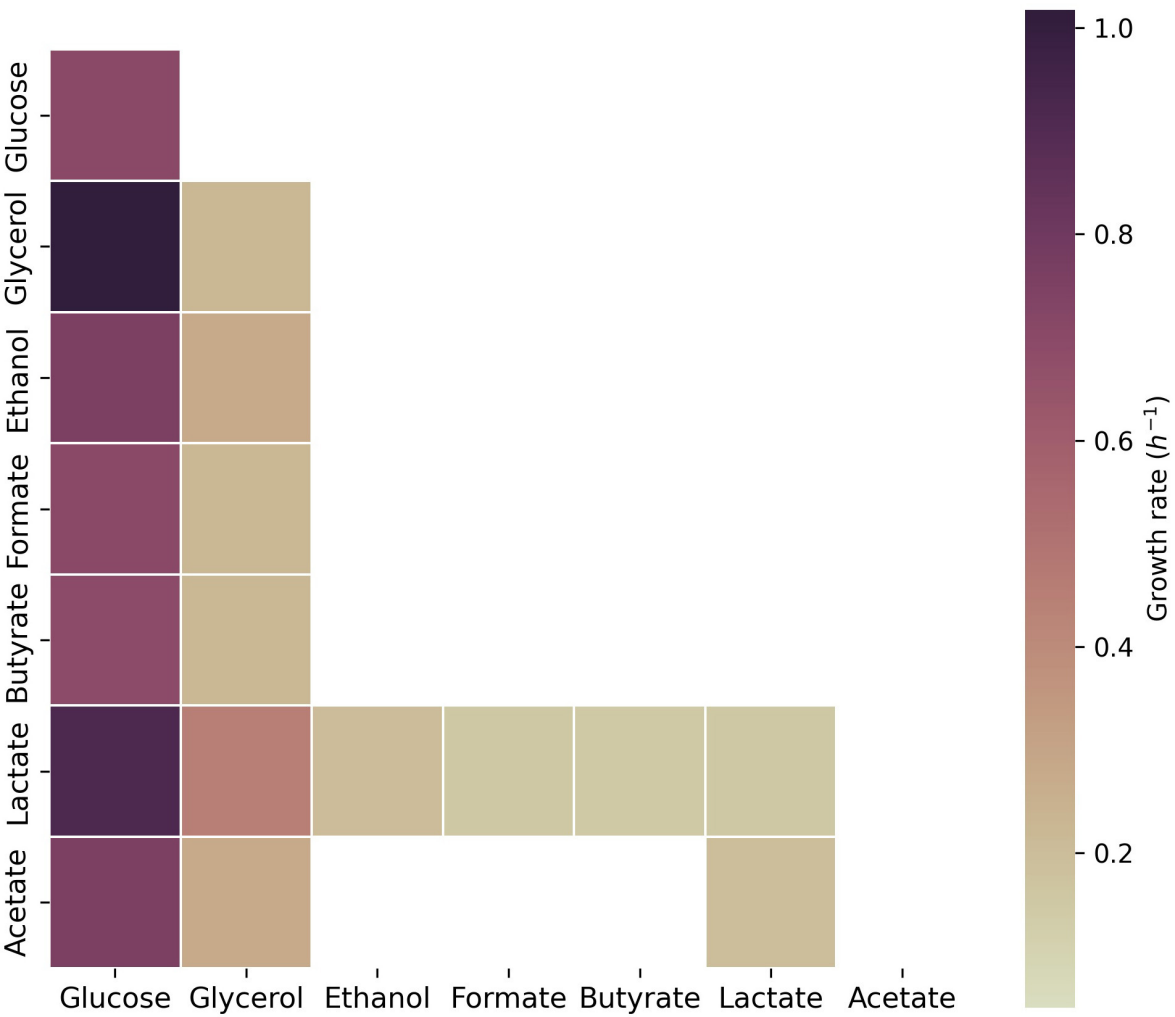
Growth capabilities predicted for *C. beijerinckii*. The color scale shows growth rates in h^-1^. Squares on the diagonal correspond to single carbon sources. Squares below the diagonal correspond to the combination of the carbon source presented on the x-axis with the carbon source presented on the y-axis. Non-colored squares show that no growth was predicted for the specified carbon source(s). The maximum uptake rate for each carbon source was set to 20 mmol g_DW_^-1^ h^-1^ and the minimum to 0.1 mmol g_DW_^-1^ h^-1^.

Additionally, we assessed growth on glucose, glycerol, ethanol, formate, butyrate, lactate, and acetate in the model of *C. beijerinckii* (Figure 3). As observed for *C. acetobutylicum, C. beijerinckii* only sustained growth on glucose, glycerol, and lactate as single carbon sources. Pairwise combinations of the latter carbon sources sustained growth in combination with the rest of carbon sources. The highest growth rates were obtained with glucose in combination with glycerol or lactate. Here, acetate with lactate or glycerol also sustained growth, being the growth rate higher with addition of acetate in both scenarios.

### 3.3. Growth of *C. acetobutylicum* and *C. beijerinckii* on lactate and acetate

**Figure 4:**
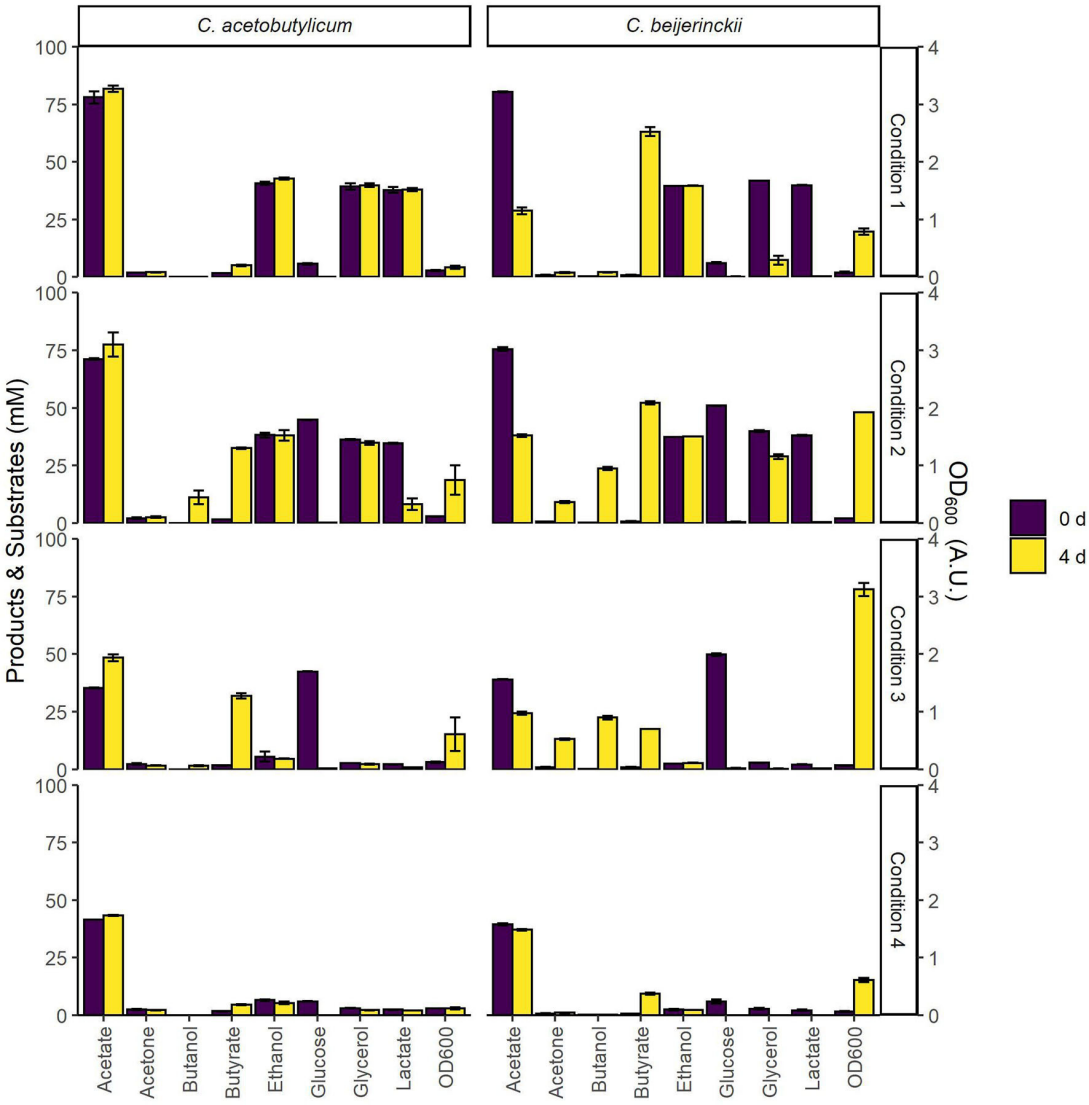
Initial screening of *C. beijerinckii* and *C. acetobutylicum* for growth on various combinations of carbon sources. The bars indicate substrate and product concentrations, and cell density at the time of inoculation and after 4 days. CM2 medium, containing 38 mM acetate, was supplemented with 40 mM of each of the various carbon sources as follows: Condition 1: acetic acid, ethanol, glycerol, and l-lactic acid; Condition 2: acetic acid, ethanol, glycerol, l-lactic acid and glucose; Condition 3: glucose. Condition 4: none. Error bars show standard deviations between two cultures inoculated with the same inoculum.

The substrate space provided by the model was used in an initial screening to assess growth and co-assimilation of acetate on various carbon sources by the solventogens *C. acetobutylicum* and *C. beijerinckii* (Figure 4). On all assessed carbon sources *C. beijerinckii* grew to higher cell densities after 4 days than *C. acetobutylicum*, which is known to produce autolysins toward the end of the exponential growth phase (Croux et al., 1992). As predicted by the models (Figures 2, 3), neither strain grew on acetate alone (Figure 4; Condition 4), as both strains only converted the residual metabolites from the inoculum, i.e., glucose. This showed the need for an additional carbon source to co-assimilate acetate. Contrary to the model predictions (Figure 2), *C. acetobutylicum* did not grow on acetate with lactate, glycerol and ethanol as co-substrates under the tested conditions. However, the model predictions for *C. beijerinckii* (Figure 3) were confirmed, and acetate was co-assimilated using all lactate and part of the glycerol into butyrate (Figure 4; Condition 1). Both solventogens further reduced butyrate to butanol with the addition of glucose (Figure 4; Condition 2). The fraction of carbon coming from acetate in the products produced by *C. beijerinckii* was not improved by the addition of glucose to the medium, and was highest in the medium containing only acetate, ethanol, glycerol, and lactate (Figure 4; Condition 1–3, Supplementary Table 1).

Growth of *C. beijerinckii* on lactate and acetate was further explored in a bioreactor at a controlled pH of 5.5 (Supplementary Figure 1). This pH is close to the optimal pH of *C. autoethanogenum* (Abrini et al., 1994), and acid re-assimilation and ABE production in *C. beijerinckii* (Diallo et al., 2020, 2021). Butyrate was the most abundant product and the stoichiometry was as follows: consumption of one mol lactate and 0.4–0.5 mol acetate produced 0.6–0.7 mol butyrate. This was similar to the stoichiometry reported for *Clostridium saccharobutylicum* NCP 262, previously known as *Clostridium acetobutylicum* P262 (Keis et al., 2001), growing on lactate and acetate (Diez-Gonzalez et al., 1995).

The screening of the solventogens *C. acetobutylicum* and *C. beijerinckii* on various carbon sources showed that the combination of *C. beijerinckii* and lactate was most promising for the re-assimilation of acetate produced from CO_2_/H_2_ by *C. autoethanogenum* in a future co-culture.

### 3.4. Fermentation of lactate and acetate by *C. beijerinckii*

**Figure 5:**
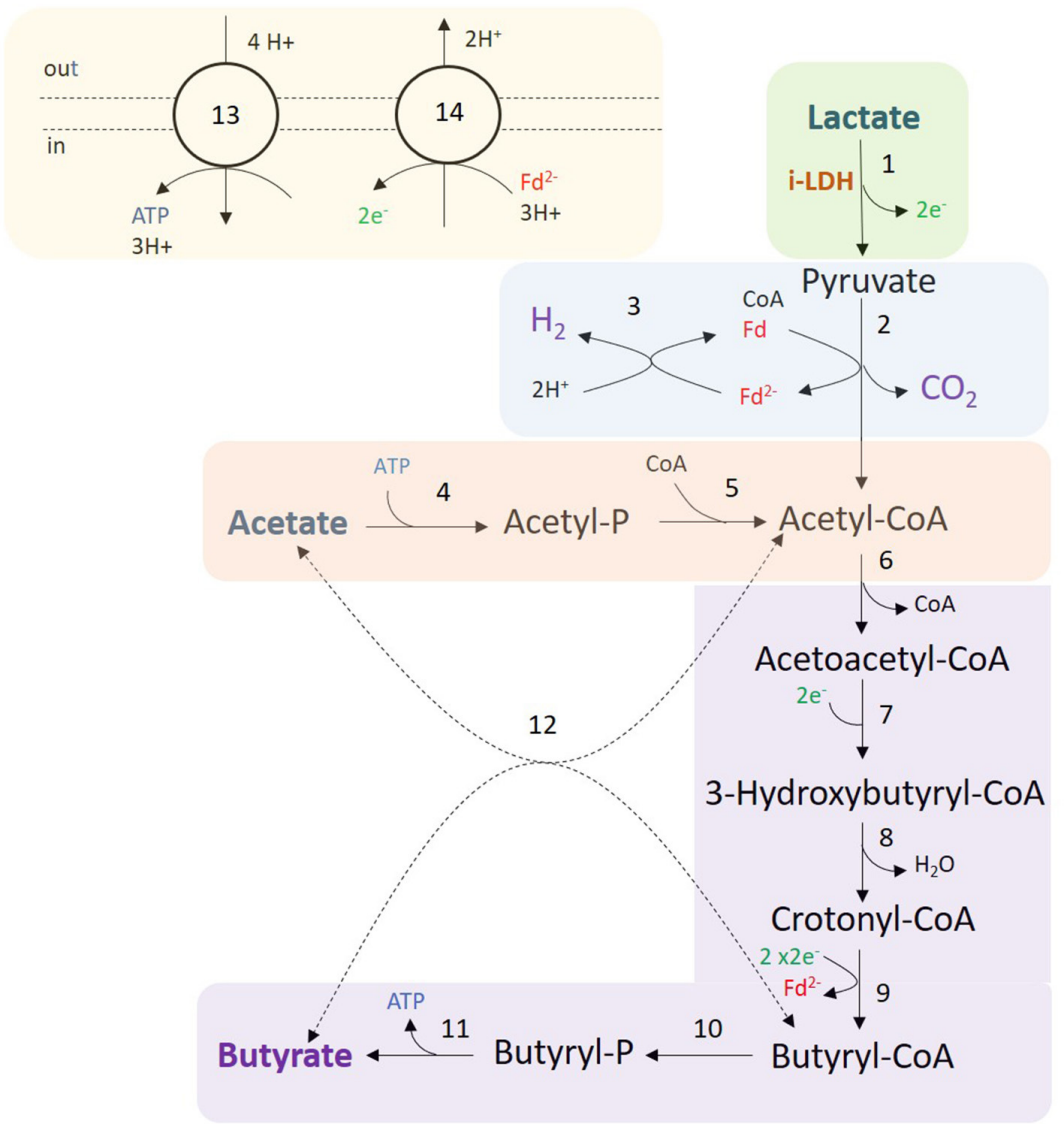
Fermentation of lactate and acetate by *C. beijerinckii*. Colored areas correspond to the following modules: lactate oxidation (green); H_2_, CO_2_, and acetyl-CoA production (blue); acetate consumption (red); butyrate production (purple); and redox cofactor regeneration, and ATPase (yellow). Numbers in reactions correspond to the following enzymes and reaction identifiers in the model: 1, NAD-independent l-lactate dehydrogenase (LDH_L); 2, pyruvate:ferredoxin oxidoreductase (POR4); 3, Ferredoxin hydrogenase (FDXNH); 4, acetate kinase (ACK); 5, phosphate acetyltransferase (PTA); 6, Acetoacetyl-CoA thiolase (ACACT1); 7, (*S*)-3-Hydroxybutanoyl-CoA:NAD^+^ oxidoreductase or NAD(P)-dependent acetoacetyl-CoA reductase (HACD1x or HACD1y); 8, 3-hydroxybutyryl-CoA dehydratase (3HBCD); 9, Butyryl-CoA dehydrogenase/electron-transferring flavoprotein complex (Bcd-EtfAB) (ACOAD1); 10, Butanoyl-CoA:phosphate butanoyltransferase (BCOPBT); 11, Butyrate kinase (BUTK); 12, Butyryl-CoA-acetoacetate CoA-transferase (COAT2); 13, ATPase (ATPase); 14, Na^+^-translocating ferredoxin:NAD^+^ oxidoreductase complex (Rnf). Dashed lines (reaction 12) indicate that the reaction might not be the main pathway.

In *C. beijerinckii*, lactate is oxidized to pyruvate *via* NAD-independent l-lactate dehydrogenase (EC 1.1.1.27) encoded by the following isoenzymes: Cbei_4072, Cbei_4903, or Cbei_2789 (Figure 5). Pyruvate is decarboxylated to acetyl-CoA *via* pyruvate:ferredoxin oxidoreductase (PFOR; EC 1.2.7.10, encoded by Cbei_1853, Cbei_4318, or Cbei_1458), generating reduced ferredoxin and CO_2_. Model predictions showed that reduced ferredoxin is partly spent to produce H_2_ and oxidized ferredoxin *via* ferredoxin hydrogenase (EC 1.12.7.2, Cbei_0327, Cbei_4000, or Cbei_3796), and partly spent to regenerate oxidized ferredoxin and NADH *via* the Rnf complex (EC 7.2.1.2, Cbei_2449-55), translocating Na^+^/H^+^ (Patakova et al., 2019). The Rnf complex is coupled to an ATPase (EC 7.1.2.2) encoded by the cluster Cbei_0412 to Cbei_0419, that pumps in Na^+^/H^+^ for energy generation.

Model predictions suggested that acetate is converted into acetyl phosphate (acetyl-P) investing ATP by acetate kinase (EC 2.7.2.1, Cbei_1165), and acetyl-P is converted into acetyl-CoA *via* phosphate acetyltransferase (EC 2.3.1.8, Cbei_3402 or Cbei_1164). As previously mentioned, *C. beijerinckii* produces butyrate *via* acetyl-CoA (Chen and Blaschek, 1999). First, two acetyl-CoA molecules are converted into one acetoacetyl-CoA by acetoacetyl-CoA thiolase (EC 2.3.1.9, Cbei_0411 or Cbei_3630). Acetoacetyl-CoA is reduced to 3-hydroxybutyryl-CoA *via* (*S*)-3-Hydroxybutanoyl-CoA:NAD^+^ oxidoreductase (EC 1.1.1.157, Cbei_0325) or *via* NAD(P)-dependent acetoacetyl-CoA reductase (EC 1.1.1.36, Cbei_5834). Then, 3-hydroxybutyryl-CoA is converted into crotonyl-CoA by 3-hydroxybutyryl-CoA dehydratase (EC 4.2.1.55, Cbei_2034 or Cbei_4544). Crotonyl-CoA is reduced *via* the butyryl-CoA dehydrogenase/electron-transferring flavoprotein complex (Bcd-EtfAB) producing reduced ferredoxin. Two complete clusters were identified in the genome: Cbei_0322 (Bcd), Cbei_0323 (EtfB), and Cbei_0324 (EtfA) or Cbei_2035 (Bcd), Cbei_2036 (EtfB) and Cbei_2037 (EtfA). An acyl-CoA dehydrogenase (Acd) showed 79.4% similarity with the Bcd subunit of *C. acetobutylicum* ATCC 824. Butyrate can be produced from butyryl-CoA *via* two routes in *C. beijerinckii*. The first route is a linear pathway in which butyryl-CoA is first converted into butyryl phosphate *via* butanoyl-CoA:phosphate butanoyltransferase (Ptb; EC 2.3.1.19, Cbei_0203). Butyryl phosphate is then converted into butyrate producing ATP *via* butyrate kinase (Buk; EC 2.7.2.7, Cbei_0204). The second route is catalyzed by a butyryl-CoA-acetoacetate CoA-transferase (EC 2.8.3.9, Cbei_2654 or Cbei_2653 or Cbei_3834 or Cbei_3833 or Cbei_4614 or Cbei_4612), where the CoA moiety of butyryl-CoA is transferred to acetate producing acetyl-CoA and butyrate. However, model predictions showed that butyrate is mostly produced generating ATP *via* Ptb and Buk.

### 3.5. Community model simulations of *C. autoethanogenum* and *C. beijerinckii* for the fermentation of CO_2_/H_2_ and lactate

The GEM of the co-culture consisted of 2,005 metabolites, 2,107 reactions, and 1,659 genes. Community model simulations supported the co-existence of the co-culture of *C. autoethanogenum* and *C. beijerinckii* for the fermentation of CO_2_/H_2_ and lactate in a wide range of growth rate and species ratio combinations.

**Figure 6:**
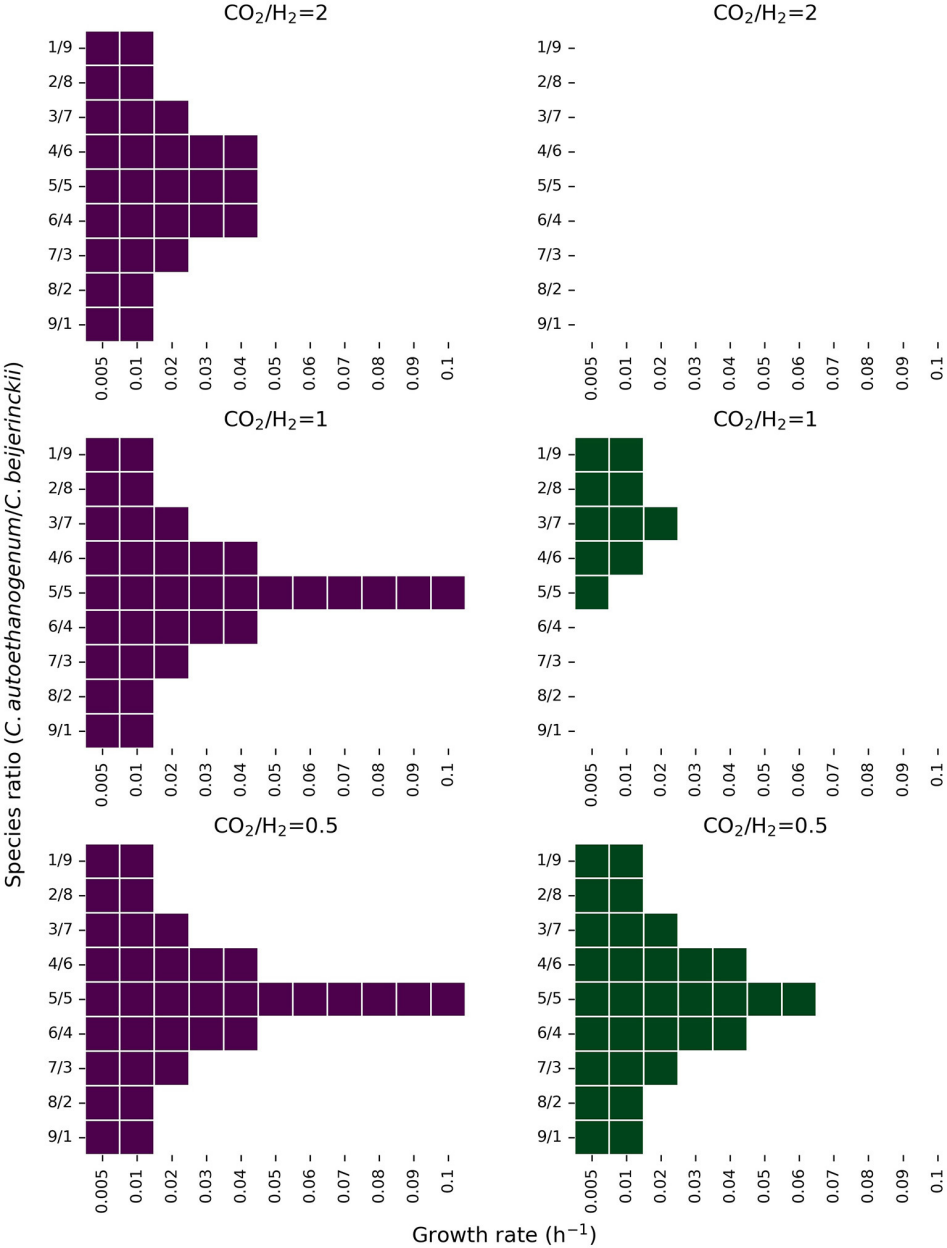
Feasible solution space of the co-culture of *C. autoethanogenum* and *C. beijerinckii* for several species ratio and growth rate combinations under different CO_2_, H_2_, and lactate feed rates. The y-axis shows the biomass species ratio of *C. autoethanogenum*/*C. beijerinckii* and the x-axis shows the growth rate in h^-1^. Colored areas indicate feasible solutions predicted by the model. Figures in purple and green show results when the lactate feed rate is set to a maximum of 5 mmol l^-1^ h^-1^, and 2.5 mmol l^-1^ h^-1^, respectively. Predictions shown on the first row were obtained with a CO_2_ and H_2_ feed rate of 5, and 2.5 mmol l^-1^ h^-1^, respectively. Predictions shown on the second row were obtained with a CO_2_ and H_2_ feed rate of 5 mmol l^-1^ h^-1^, and on the third row, with a CO_2_ and H_2_ feed rate of 2.5, and 5 mmol l^-1^ h^-1^, respectively.

Figure 6 shows the feasible solution space for multiple combinations of species ratios, growth rates, CO_2_, H_2_, and lactate feeds. When the maximum uptake rate of lactate is 2.5 mmol l^-1^ h^-1^ (Figure 6; green figures), the feasibility of the co-culture becomes more limited. In these conditions, the co-culture is only feasible at low growth rates (<0.02 h^-1^) for all species ratios, and feasible at higher growth rates (up to 0.07 h^-1^) when *C. autoethanogenum* and *C. beijerinckii* are similarly present in the community for CO_2_/H_2_ feed ratio of 0.5. The co-culture is infeasible in all conditions when the CO_2_/H_2_ feed ratio is 2, and only feasible when the presence of *C. autoethanogenum* is low and the CO_2_/H_2_ feed ratio is 1. The range of feasible solutions becomes wider when the lactate feed rate is 5 mmol l^-1^ h^-1^. When *C. autoethanogenum* and *C. beijerinckii* are equally present in the community, the co-culture can be established with all explored growth rates, except when the CO_2_/H_2_ feed ratio is 2, that is only feasible up to 0.04 h^-1^. Again, only at lower growth rates (<0.02 h^-1^), the co-culture is feasible for all tested species ratios.

**Figure 7:**
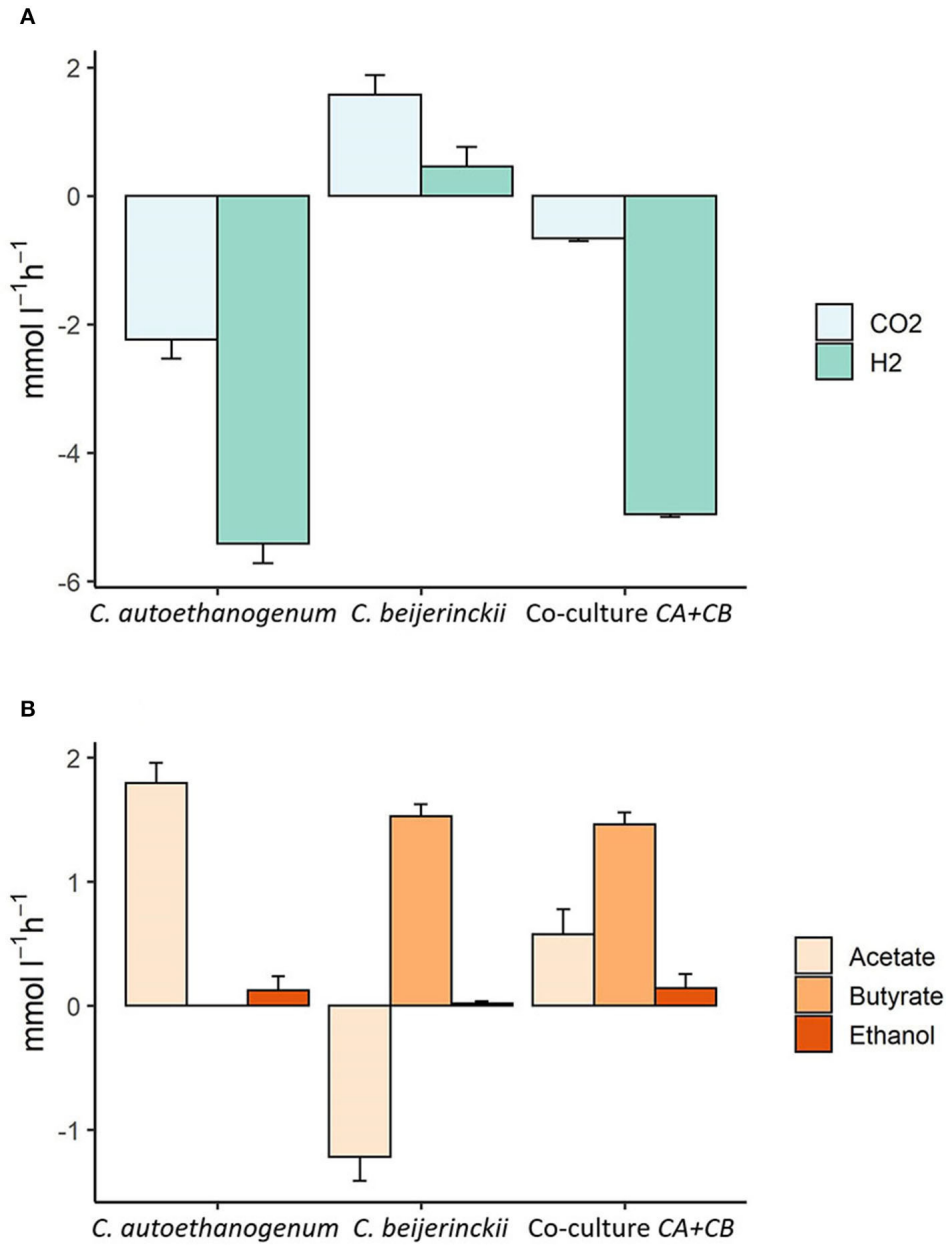
Steady-state production and consumption rates of the main substrates and products predicted by the community model of *C. autoethanogenum* and *C. beijerinckii*. The x-axis shows the species associated to the illustrated fluxes. The y-axis shows uptake (negative) or production (positive) fluxes in mmol l^-1^ h^-1^. **(A)** Shows CO_2_ and H_2_ production or consumption rates, and **(B)** shows the production or uptake of acetate, butyrate, and ethanol. Modeled uptake and production rates are shown for *C. autoethanogenum, C. beijerinckii* and for the co-culture of *C. autoethanogenum* and *C. beijerinckii* (Co-culture *CA+CB*), respectively. Growth rate was set to 0.02 h^-1^; biomass species ratio was set to 1:1; maximum and minimum lactate uptake rate was set to 2.5 and 0.1 mmol l^-1^ h^-1^, and the maximum and minimum uptake of CO_2_ and H_2_ were set to 5 and 0.5 mmol l^-1^ h^-1^, respectively.

Figure 7 shows the steady-state consumption and production rates observed in co-culture compared to the consumption and production rates associated to *C. autoethanogenum* or *C. beijerinckii*. Part of the acetate produced by *C. autoethanogenum* is taken up by *C. beijerinckii* since the steady-state production rates in the co-culture are lower than the production rates of *C. autoethanogenum*. The fermentation of acetate and lactate leads to the production of butyrate in *C. beijerinckii*. A small amount of butyrate is reassimilated by *C. autoethanogenum* and by *C. beijerinckii* and converted into butanol (not shown). Furthermore, ethanol is being produced in smaller amounts by *C. autoethanogenum* and *C. beijerinckii*. Model predictions also showed an exchange of CO_2_ and H_2_ from *C. beijerinckii* to *C. autoethanogenum*. *C. beijerinckii* produces CO_2_ and H_2_ that are taken up by *C. autoethanogenum*, since the flux through *C. autoethanogenum* is higher than the flux through the exchange reaction in the co-culture. Model predictions suggested that acetate consumption by *C. beijerinckii* varied depending on the lactate feed rate, being lower when the lactate feed rate was higher than 2.5 mmol l^-1^ h^-1^ (≈ 5 mmol l^-1^ h^-1^). A higher lactate feed rate also led to more CO_2_ and H_2_ produced by *C. beijerinckii*, and thus, to more gases being recirculated and consumed by *C. autoethanogenum* producing more acetate (git repository).

Furthermore, we observed traces of formate, 2,3-butanediol, acetone, isopropanol, and butanol (see git repository).

### 3.6. Fed-batch fermentation of CO_2_/H_2_ and lactate by the novel co-culture of *C. autoethanogenum* and *C. beijerinckii*

**Figure 8:**
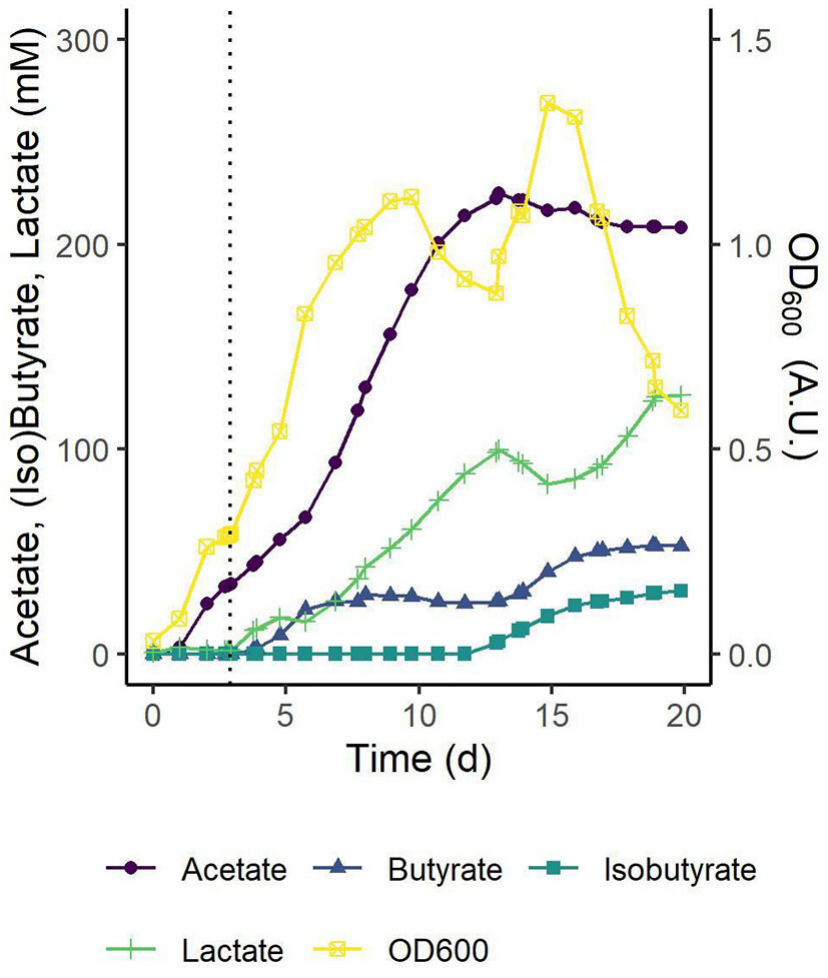
pH-controlled fed-batch fermentation of the *C. autoethanogenum*–*C. beijerinckii* co-culture on 1:4 CO_2_/H_2_ with an l-lactic acid feed. Concentrations of the main substrates, products, and cell density are shown. At t_0_ cultures were inoculated with *C. autoethanogenum*. The dotted black line marks inoculation with *C. beijerinckii* and the start of the l-lactic acid feed. l-lactic acid was fed at a rate of 3 ml d^-1^ till 19 d. Traces of ethanol (3–8 d, max. 4.6 mM at 7 d and 13–20 d, max. 5.6 mM at 20 d), butanol (14–20 d, max. 3.1 mM at 20 d) and glucose from the *C. beijerinckii* inoculum (<1 mM at 3 d) were detected. pH was controlled at pH 5.5 ± 0.1. Results of a single biological replicate are shown here and the results of a second independent biological replicate are shown in Supplementary Figure 2.

Production of butyrate from CO_2_/H_2_ and the co-substrate lactate by the modeled co-culture of *C. autoethanogenum* and *C. beijerinckii* was experimentally verified with two biologically independent pH-controlled fed-batch fermentations (Figure 8 and Supplementary Figure 2). Both fermentations showed similar trends in biomass production and metabolites profiles. Below, the results are described for the fermentation shown in Figure 8. Initially, *C. autoethanogenum* was grown solely on a continuous CO_2_/H_2_ feed, and after 3 d, the OD_600_ had reached a value of 0.29 and the acetate concentration a value of 34 mM. This acetate concentration was considered sufficient to support *C. beijerinckii*. *C. beijerinckii* was added and the l-lactic acid feed was started. The rate of the l-lactic acid feed was set lower than the rate of acetate production from CO_2_/H_2_ by *C. autoethanogenum* to prevent complete depletion of acetate. Upon inoculation with *C. beijerinckii* and the start of the l-lactic acid feed, a continued growth phase was observed till 10 d in which butyrate was produced up to 28 mM.

A theoretical acetate production from CO_2_ was calculated from which the corresponding stoichiometry for butyrate production at each time point was calculated (Supplementary Figure 2). Between 4 and 7 d, during butyrate production in the first growth phase, for each mol of consumed lactate, 0.2–1 mol acetate was reassimilated, and 0.5–0.6 mol butyrate was produced.

The drop in cell density observed between 10 and 13 d could be explained by the accumulation of biomass observed at the reactor wall above the fermentation medium from 6 d onward (data not shown). No production of butyrate was observed in this period and microscope observations showed that the consortium consisted almost entirely of vegetative cells (data not shown). These cells could not be assigned to either species as the morphologies of *C. autoethanogenum* and *C. beijerinckii* could not be clearly distinguished. As a result, the species ratio in the co-culture was not determined experimentally. In future studies, the species ratio could be obtained from transcriptomic (Benito-Vaquerizo et al., 2020), amplicon (Ibarbalz et al., 2014), or qPCR data (Charubin and Papoutsakis, 2019).

After this adaptation period, a second growth phase was observed between 13 and 15 d coinciding with a larger fraction of sporulating cells in the culture and the co-production of butyrate and isobutyrate to final concentrations of 53 and 31 mM, respectively. While measured in both replicates (Figure 8 and Supplementary Figure 2), the production of isobutyrate by the consortium was not predicted by the models, and will be further investigated in a follow-up research. The calculated stoichiometry indicated a shift toward the conversion of lactate during this second growth and production phase (Supplementary Figure 2).

### 3.7. Analysis of substrate consumption and product formation by the co-culture model

**Figure 9:**
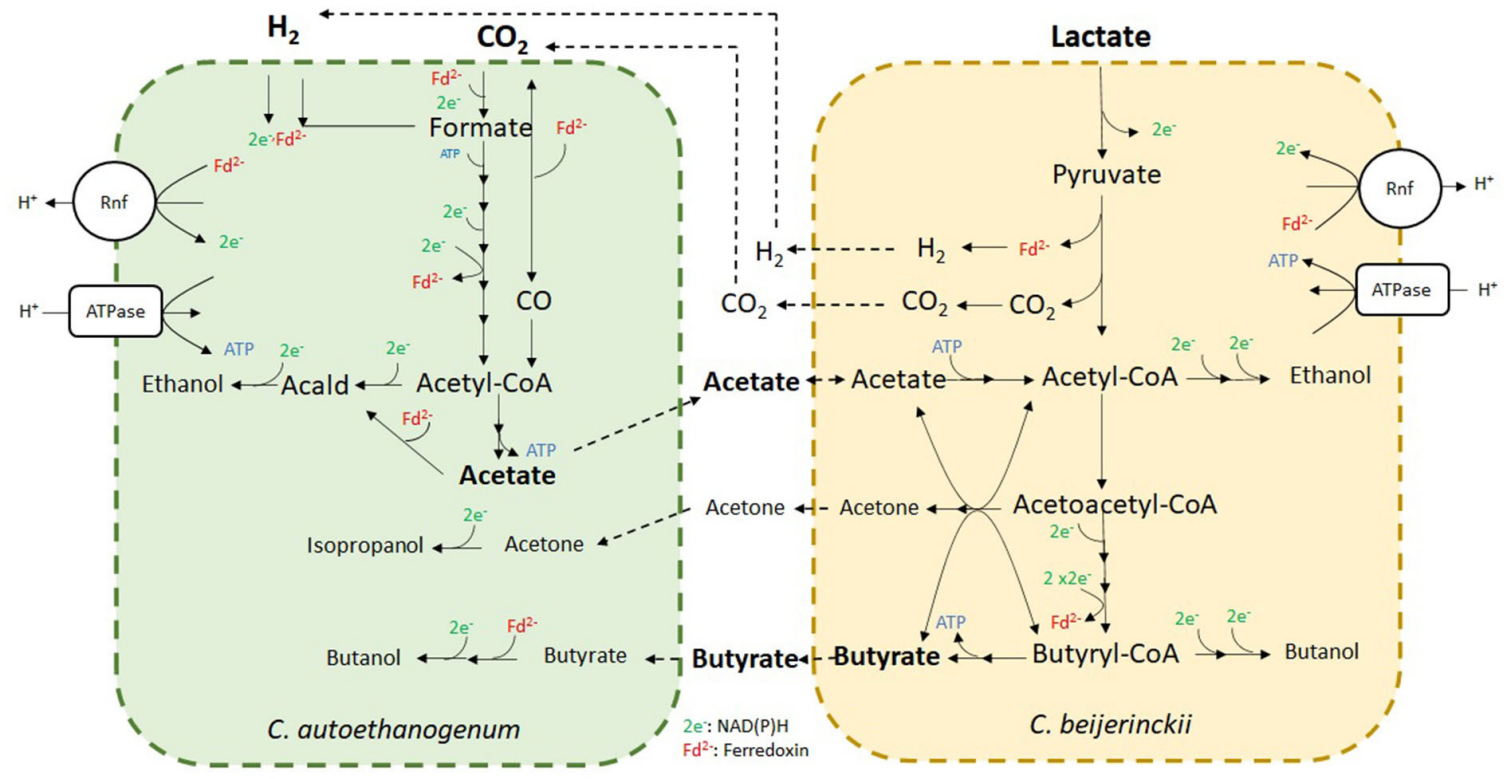
Fermentation of CO_2_/H_2_ and lactate by the novel co-culture of *C. autoethanogenum* and *C. beijerinckii*. Metabolites in bold indicate substrates and main products. Metabolites in smaller letter size indicate minor products. Arrows indicate the flux direction. Dashed lines display transport reactions of metabolites from the extracellular compartment to the intracellular compartment of the indicated microbe, and *vice versa*.

*C. autoethanogenum* takes-up CO_2_ and H_2_ through the Wood-Ljungdahl pathway, where H_2_ is used as an electron donor for CO_2_ reduction to acetyl-CoA (Figure 9). Acetyl-CoA is mainly converted into acetate producing ATP, and ethanol. In addition, traces of 2,3-butanediol, formate and lactate were predicted by the model (not shown here). Part of the acetate was in turn taken-up by *C. beijerinckii* together with the external lactate feed, following the metabolism described in Section 3.3. Ethanol was produced by *C. autoethanogenum* and by *C. beijerinckii*, as observed in the experiments. Model simulations suggested the production of butyrate, CO_2_ and H_2_ by *C. beijerinckii*, and traces of acetone and butanol. We observed that most of the CO_2_ and H_2_ produced by *C. beijerinckii* was metabolized by *C. autoethanogenum* (Figure 8). Furthermore, we observed traces of isopropanol formed from the conversion of the assimilated acetone by *C. autoethanogenum* through an alcohol dehydrogenase. The community model suggested that butanol was produced by *C. beijerinckii* and by *C. autoethanogenum* (Figure 9). As Diender et al. (2016) already observed, butyrate could be exchanged between *C. beijerinckii* and *C. autoethanogenum*, and converted into butanol by an alcohol dehydrogenase and the aldehyde ferredoxin oxidoreductase.

In addition, the model predicts traces of formate and lactate produced by *C. autoethanogenum* being assimilated by *C. beijerinckii* (not shown here).

Supplementary Figure 3 represents the metabolic profile of the novel co-culture with glucose, instead of lactate, as additional carbon source. As observed in Figure 4, the addition of glucose would lead to an increase of ABE production by *C. beijerinckii*, since there are more reducing equivalents when glucose is converted to pyruvate. Acetate would still be the main product in *C. autoethanogenum* and ethanol would be produced in minor amounts. Part of the acetate could be metabolized by *C. beijerinckii*, but also produced together with butyrate during the acidogenic phase. Once the pH drops enough, the acids could be partly reassimilated during solventogensis to produce solvents. Acetone and butyrate could be partly taken up by *C. autoethanogenum* producing isopropanol and more butanol. Possibly, part of the CO_2_ and H_2_ produced by *C. beijerinckii* would be consumed by *C. autoethanogenum* as described for the co-culture growing on lactate.

## 4. Discussion

GEMs are mathematical representations of the metabolism and have been successfully employed to gain insights into metabolic capabilities of single species (Milne et al., 2011; Dash et al., 2014; Valgepea et al., 2017; Gu et al., 2019; Benito-Vaquerizo et al., 2022), and to elucidate possible strategies to optimize the performance of microorganism(s) in mono- and co-cultivation (Kim et al., 2019; Benito-Vaquerizo et al., 2020; Foster et al., 2021; García-Jiménez et al., 2021). In this study, the use of constrained-based modeling has been key to design an alternative way to produce butyrate from CO_2_/H_2_ and lactate. We have proven the capacity of *C. beijerinckii* NCIMB 8052 to grow on lactate and acetate as the sole carbon and energy source. Moved by the need to upcycle sustainable feedstocks, we have used this new found capacity of *C. beijerinckii* to established a novel synthetic co-culture of *C. autoethanogenum* and *C. beijerinckii* for the fermentation of CO_2_/H_2_ and lactate into butyrate.

The use of lactate as alternative carbon source by *C. beijerinckii* as co-substrate with acetate creates new possibilities for the production of butyrate. Acetate is the most abundant product of gas fermentation, and therefore, has an essential role in the establishment of the co-culture. Lactate is a minor fermentation product of acetogens grown on syngas or CO_2_/H_2_ (Valgepea et al., 2018; Im et al., 2022), but a major fermentation product of acetogens grown on sugars (Drake and Daniel, 2004). Acetogens could be engineered toward autotrophic lactate production from CO_2_/H_2_ (Mook et al., 2022) or syngas, thereby facilitating butyrate production in co-cultivation with *C. beijerinckii* without the need of adding an additional carbon source. Alternatively, lactate can be obtained from other sources, such as side-streams from the dairy industry (Sar et al., 2022), spoiled agri-food products (Xu et al., 2020), ensiled agricultural biomass (Chen et al., 2007), and fermented grass (Sakarika et al., 2022).

*C. beijerinckii* has wide physiological versatility, which makes this microbe an ideal candidate to produce butyrate in a co-culture. However, butyrate production could also be achieved by the co-cultivation of an acetogen with a butyrate producing species, such as *Clostridium butyricum*, whose ability to grow on lactate and acetate was also proved recently (Detman et al., 2019).

Additionally, the use of the newly established co-culture could increase carbon recycling and electron transport, since model predictions indicated that CO_2_ and H_2_ produced by *C. beijerinckii* were almost fully reassimilated by *C. autoethanogenum* (Figure 7), which reduces the carbon footprint. Incorporation of an organism able to produce H_2_ needed for CO_2_ assimilation is interesting to consider for future approaches. Besides solventogenic Clostridia, other anaerobic bacterial species have been described that produce H_2_ from the fermentation of sugars at high yields (Show et al., 2012). This opens up new alternatives for more efficient co-cultures without the need of external H_2_.

Model predictions showed slow growth on lactate and acetate by *C. acetobutylicum*. However, this was not confirmed by experiments in which lactate was not consumed, and acetate was produced rather than consumed (Figure 4; condition 1). Diez-Gonzalez et al. (1995) showed growth on lactate and acetate in the solventogen *C. saccharobutylicum* NCP 262. They analyzed extracts of cells grown on lactate and acetate and observed NAD-dependent lactate dehydrogenase (d-LDH) as well as NAD-independent lactate dehydrogenase (i-LDH) activity. d-LDH regulated the conversion of pyruvate to lactate and required fructose-1,6-biphosphate to be active (Freier and Gottschalk, 1987; Diez-Gonzalez et al., 1995). i-LDH regulated the conversion of lactate to pyruvate (Figure 5) and had double the activity over d-LDH. In addition, i-LDH activity decreased fourfold when glucose was added to cultures growing on lactate and acetate. However, lactate was only converted by *C. acetobutylicum* ATCC 824 when glucose was added (Figure 4; Condition 2) suggesting that i-LDH from *C. acetobutylicum* ATCC 824 is activated by glucose. Interestingly, the LDH of *C. beijerinckii* NCIMB 8052 and *C. acetobutylicum* ATCC 824 showed 87.7 and 57% similarity with the LDH of *C. saccharobutylicum* NCP 262, respectively. Keis et al. (2001) showed that *C. saccharobutylicum* NCP 262 is more similar to *C. beijerinckii* NCIMB 8052 than to *C. acetobutylicum* ATCC 824. Therefore, we hypothesize that *C. beijerinckii* has an i-LDH activity comparable to *C. saccharobutylicum*, whereas i-LDH activity in *C. acetobutylicum* ATCC 824 is regulated differently.

Model predictions showed a high production of butyrate, acetate, and traces of ethanol, acetone, butanol, isopropanol, 2,3-butanediol, and formate. Fed-batch experiments also showed butyrate and acetate as major fermentations products, and ethanol and butanol as minor fermentation products. Charubin and Papoutsakis (2019) observed production of 2,3-butanediol from the assimilation by *C. ljungdahlii* of the acetoin produced by *C. acetobutylicum*. However, acetolactate decarboxylase was only annotated in the genome of *C. autoethanogenum* and not in the genome of *C. beijerinckii* (Siemerink et al., 2014), and thus, acetoin could not be produced by the solventogen. Lactate degradation results in less NAD(P)H available, and therefore, the production of solvents is lower compared to the standard ABE fermentation on sugars (Sreekumar et al., 2015; Charubin and Papoutsakis, 2019). In contrast, the co-culture in our study has a relatively high butyrate production (up to 53 mM). Furthermore, mono-culture experiments on lactate and acetate in our study produced higher concentrations of butyrate (up to 42 mM) than the reported co-assimilation of glycerol and acetate by *C. beijerinckii* (≈20 mM) (Kumar et al., 2022), and than the co-assimilation of lactate and acetate by *C. saccharobutylicum* (Diez-Gonzalez et al., 1995) (≈20 mM).

Model predictions indicated that *C. beijerinckii* could grow on lactate as the sole carbon and energy source, as was recently observed (Schwalm et al., 2019). However, the growth rate was improved by the addition of acetate (Figure 3), as was previously shown (Diez-Gonzalez et al., 1995). The addition of acetate favors lactate uptake, since the acetyl-CoA pool increases with addition of acetate as co-substrate, and thus, more acetyl-CoA would be converted into butyrate producing more ATP. Co-culture fed-batch experiments showed, however, accumulation of acetate in the fermentation broth. This showed that not all acetate produced by *C. autoethanogenum* was consumed by *C. beijerinckii*, as indicated by the model, and possibly that some acetate could also be produced by *C. beijerinckii*.

We should note that the deployed modeling approach predicts steady-state production or consumption rates, and thus, we cannot compare the results quantitatively with bioreactor data, which consist of concentrations over time. Instead, our study should be seen as an exploratory study assessing the feasibility of the co-culture. Future optimization of this co-culture could integrate current experimental data and relative abundance of species into dynamic modeling approaches to gain better insights into the concentration profiles over time. These results show that community modeling of metabolism is a valuable tool to guide the design of microbial consortia for the tailored production of important chemicals from renewable resources. It thereby expands the space of options to possibly accelerate the transition to a biobased economy.

## 5. Conclusion

Genome-scale metabolic modeling helped identifying the ability of *C. beijerinckii* to co-metabolize acetate and lactate for the production of butyrate. This ability was experimentally verified in batch serum bottles and pH-controlled batch bioreactor fermentations. A community model of *C. autoethanogenum* and *C. beijerinckii* was then constructed to assess the feasibility of the co-culture to produce butyrate from CO_2_/H_2_ and lactate. Community modeling predicted the feasibility of the co-culture in several conditions and the interactions between species, especially, the exchange of acetate. Following model predictions, the co-culture of *C. autoethanogenum* and *C. beijerinckii* was established in pH-controlled fed-batch fermentations. The main products were acetate, butyrate and the newly identified metabolite, isobutyrate. Our study shows the strength of a model-driven approach to explore the high metabolic flexibility of clostridial species for the production of chemicals from renewable sources.

## Data availability statement

The original contributions presented in the study are included in the article/Supplementary material, further inquiries can be directed to the corresponding author.

## Author contributions

SB-V and NN conceived and designed the study and drafted the manuscript. SB-V constructed the models and performed model simulations and data analysis. NN performed the experiments and data analysis. MS-D, AML-C, JH, SB, PS, and VM conceived, designed, and supervised the research. MS-D, VM, JH, and AML-C acquired project funding. All authors reviewed and edited the study. All authors read and approved the content of the submitted version.

## References

[B1] Abrini J. Naveau H. Nyns E. J. (1994). *Clostridium autoethanogenum*, sp. nov., an anaerobic bacterium that produces ethanol from carbon monoxide. Arch. Microbiol. 161, 345–351. 10.1007/BF00303591

[B2] Bae J. Song Y. Lee H. Shin J. Jin S. Kang S.. (2022). Valorization of C1 gases to value-added chemicals using acetogenic biocatalysts. Chem. Eng. J. 428:131325. 10.1016/j.cej.2021.131325

[B3] Bengelsdorf F. R. Beck M. H. Erz C. Hoffmeister S. Karl M. M. Riegler P.. (2018). Bacterial anaerobic synthesis gas (syngas) and CO_2_H_2_ fermentation. Adv. Appl. Microbiol. 103, 143–221. 10.1016/bs.aambs.2018.01.002 29914657

[B4] Benito-Vaquerizo S. Diender M. Parera Olm I. Martins dos Santos V. A. Schaap P. J. Sousa D. Z.. (2020). Modeling a co-culture of *Clostridium autoethanogenum* and *Clostridium kluyveri* to increase syngas conversion to medium-chain fatty-acids. Comput. Struct. Biotechnol. J. 18, 3255–3266. 10.1101/2020.06.23.167189 33240469 PMC7658664

[B5] Benito-Vaquerizo S. Olm I. P. de Vroet T. Schaap P. J. Sousa D. Z. dos Santos V. A. M. Suarez-Diez M. (2022). Genome-scale metabolic modelling enables deciphering ethanol metabolism *via* the acrylate pathway in the propionate-producer Anaerotignum neopropionicum. Microb. Cell Fact. 21, 1–18. 10.1186/s12934-022-01841-1 35710409 PMC9205015

[B6] Bertsch J. Müller V. (2015). Bioenergetic constraints for conversion of syngas to biofuels in acetogenic bacteria. Biotechnol. Biofuels 8, 1–12. 10.1186/s13068-015-0393-x 26692897 PMC4676187

[B7] Brändle J. Domig K. J. Kneifel W. (2016). Relevance and analysis of butyric acid producing clostridia in milk and cheese. Food Control 67, 96–113. 10.1016/j.foodcont.2016.02.038

[B8] Casau M. Dias M. F. Matias J. C. Nunes L. J. (2022). Residual biomass: a comprehensive review on the importance, uses and potential in a circular bioeconomy approach. Resources 11:35. 10.3390/resources11040035

[B9] Chan S. H. J. Simons M. N. Maranas C. D. (2017). Steadycom: predicting microbial abundances while ensuring community stability. PLoS Comput. Biol. 13:e1005539. 10.1371/journal.pcbi.1005539 28505184 PMC5448816

[B10] Charubin K. Papoutsakis E. T. (2019). Direct cell-to-cell exchange of matter in a synthetic *Clostridium syntrophy* enables CO_2_ fixation, superior metabolite yields, and an expanded metabolic space. Metab. Eng. 52, 9–19. 10.1016/j.ymben.2018.10.006 30391511

[B11] Chen C. K. Blaschek H. P. (1999). Effect of acetate on molecular and physiological aspects of *Clostridium beijerinckii* NCIMB 8052 solvent production and strain degeneration. Appl. Environ. Microbiol. 65, 499–505. 10.1128/AEM.65.2.499-505.1999 9925574 PMC91053

[B12] Chen Y. Sharma-Shivappa R. R. Chen C. (2007). Ensiling agricultural residues for bioethanol production. Appl. Biochem. Biotechnol. 143, 80–92. 10.1007/s12010-007-0030-7 18025598

[B13] Croux C. Canard B. Goma G. Soucaille P. (1992). Autolysis of *Clostridium acetobutylicum* ATCC 824. J. Gen. Microbiol. 138, 861–869. 10.1099/00221287-138-5-861 1645127

[B14] Dash S. Mueller T. J. Venkataramanan K. P. Papoutsakis E. T. Maranas C. D. (2014). Capturing the response of *Clostridium acetobutylicum* to chemical stressors using a regulated genome-scale metabolic model. Biotechnol. Biofuels 7, 1–16. 10.1186/s13068-014-0144-4 25379054 PMC4207355

[B15] Detman A. Mielecki D. Chojnacka A. Salamon A. Błaszczyk M. K. Sikora A. (2019). Cell factories converting lactate and acetate to butyrate: *Clostridium butyricum* and microbial communities from dark fermentation bioreactors. Microb. Cell Factories 18, 1–12. 10.1186/s12934-019-1085-1 30760264 PMC6373154

[B16] Diallo M. Kengen S. W. López-Contreras A. M. (2021). Sporulation in solventogenic and acetogenic Clostridia. Appl. Microbiol. Biotechnol. 105, 3533–3557. 10.1007/s00253-021-11289-9 33900426 PMC8102284

[B17] Diallo M. Kint N. Monot M. Collas F. Martin-Verstraete I. Oost J. V. D.. (2020). Transcriptomic and phenotypic analysis of a spoiie mutant in *Clostridium beijerinckii*. Front. Microbiol. 11:556064. 10.3389/fmicb.2020.556064 33042064 PMC7522474

[B18] Diender M. Olm I. P. Gelderloos M. Koehorst J. J. Schaap P. J. Stams A. J.. (2019). Metabolic shift induced by synthetic co-cultivation promotes high yield of chain elongated acids from syngas. Sci. Rep. 9, 1–11. 10.1038/s41598-019-54445-y 31792266 PMC6889307

[B19] Diender M. Stams A. J. Sousa D. Z. (2016). Production of medium-chain fatty acids and higher alcohols by a synthetic co-culture grown on carbon monoxide or syngas. Biotechnol. Biofuels 9, 1–11. 10.1186/s13068-016-0495-0 27042211 PMC4818930

[B20] Diez-Gonzalez F. Russell J. B. Hunter J. B. (1995). The role of an NAD-independent lactate dehydrogenase and acetate in the utilization of lactate by *Clostridium acetobutylicum* strain P262. Arch. Microbiol. 164, 36–42. 10.1007/BF02568732

[B21] Drake H. L. Daniel S. L. (2004). Physiology of the thermophilic acetogen *Moorella thermoacetica*. Res. Microbiol. 155, 422–36. 10.1016/j.resmic.2004.03.003 15249059

[B22] Du Y. Zou W. Zhang K. Ye G. Yang J. (2020). Advances and applications of *Clostridium* co-culture systems in biotechnology. Front. Microbiol. 11:2842. 10.3389/fmicb.2020.560223 33312166 PMC7701477

[B23] Dwidar M. Park J. Y. Mitchell R. J. Sang B. I. (2012). The future of butyric acid in industry. Sci. World J. 2012:471417. 10.1100/2012/471417 22593687 PMC3349206

[B24] Ebrahim A. Lerman J. A. Palsson B. O. Hyduke D. R. (2013). COBRApy: constraints-based reconstruction and analysis for python. BMC Syst. Biol. 7:74. 10.1186/1752-0509-7-74 23927696 PMC3751080

[B25] Foster C. Charubin K. Papoutsakis E. T. Maranas C. D. (2021). Modeling growth kinetics, interspecies cell fusion, and metabolism of a *Clostridium acetobutylicum/Clostridium ljungdahlii* syntrophic coculture. mSystems 6:e01325-20. 10.1128/mSystems.01325-20 33622858 PMC8573953

[B26] Freier D. Gottschalk G. (1987). L(+)-lactate dehydrogenase of *Clostridium acetobutylicum* is activated by fructose-1,6-bisphosphate. FEMS Microbiol. Lett. 43, 229–233. 10.1111/j.1574-6968.1987.tb02128.x

[B27] García-Jiménez B. Torres-Bacete J. Nogales J. (2021). Metabolic modelling approaches for describing and engineering microbial communities. Comput. Struct. Biotechnol. J. 19:226. 10.1016/j.csbj.2020.12.003 33425254 PMC7773532

[B28] Gottinger A. Ladu L. Quitzow R. (2020). Studying the transition towards a circular bioeconomy-a systematic literature review on transition studies and existing barriers. Sustainability 12:8990. 10.3390/su12218990

[B29] Gu C. Kim G. B. Kim W. J. Kim H. U. Lee S. Y. (2019). Current status and applications of genome-scale metabolic models. Genome Biol. 20, 1–18. 10.1186/s13059-019-1730-3 31196170 PMC6567666

[B30] Hwang H. W. Yoon J. Min K. Kim M. S. Kim S. J. Cho D. H.. (2020). Two-stage bioconversion of carbon monoxide to biopolymers *via* formate as an intermediate. Chem. Eng. J. 389:124394. 10.1016/j.cej.2020.124394

[B31] Ibarbalz F. M. Pérez M. V. Figuerola E. L. M. Erijman L. (2014). The bias associated with amplicon sequencing does not affect the quantitative assessment of bacterial community dynamics. PLoS ONE 9:e99722. 10.1371/journal.pone.0099722 24923665 PMC4055690

[B32] Im C. Valgepea K. Modin O. Nygård Y. (2022). *Clostridium ljungdahlii* as a biocatalyst in microbial electrosynthesis-Effect of culture conditions on product formation. Bioresour. Technol. Rep. 19:101156. 10.1016/j.biteb.2022.101156

[B33] Keis S. Sullivan J. T. Jones D. T. (2001). Physical and genetic map of the *Clostridium saccharobutylicum* (formerly *Clostridium acetobutylicum*) NCP 262 chromosome. Microbiology 147, 1909–1922. 10.1099/00221287-147-7-1909 11429467

[B34] Khandelwal R. A. Olivier B. G. Röling W. F. M. Teusink B. Bruggeman F. J. (2013). Community flux balance analysis for microbial consortia at balanced growth. PLoS ONE 8:e64567. 10.1371/journal.pone.0064567 23741341 PMC3669319

[B35] Kim M. Park B. G. Kim E. J. Kim J. Kim B. G. (2019). *In silico* identification of metabolic engineering strategies for improved lipid production in *Yarrowia lipolytica* by genome-scale metabolic modeling. Biotechnol. Biofuels 12, 1–14. 10.1186/s13068-019-1518-4 31367232 PMC6657051

[B36] Köpke M. Gerth M. L. Maddock D. J. Mueller A. P. Liew F. M. Simpson S. D.. (2014). Reconstruction of an acetogenic 2,3-butanediol pathway involving a novel NADPH-dependent primary-secondary alcohol dehydrogenase. Appl. Environ. Microbiol. 80:3394. 10.1128/AEM.00301-14 24657865 PMC4018851

[B37] Kremp F. Roth J. Müller V. (2020). The sporomusa type Nfn is a novel type of electron-bifurcating transhydrogenase that links the redox pools in acetogenic bacteria. Sci. Rep. 10, 1–14. 10.1038/s41598-020-71038-2 32913242 PMC7483475

[B38] Kuit W. Minton N. P. López-Contreras A. M. Eggink G. (2012). Disruption of the acetate kinase (ACK) gene of *Clostridium acetobutylicum* results in delayed acetate production. Appl. Microbiol. Biotechnol. 94:729. 10.1007/s00253-011-3848-4 22249720 PMC3315644

[B39] Kumar E. Gangwar S. Ujor B. Glycerol V. C. Agyeman-Duah E. Kumar S.. (2022). Glycerol utilization as a sole carbon source disrupts the membrane architecture and solventogenesis in *Clostridium beijerinckii* NCIMB 8052. Fermentation 8:339. 10.3390/fermentation8070339

[B40] Lee H. Bae J. Jin S. Kang S. Cho B.-K. (2022). Engineering acetogenic bacteria for efficient one-carbon utilization. Front. Microbiol. 13:865168. 10.3389/fmicb.2022.865168 35615514 PMC9124964

[B41] Liao C. Seo S. O. Celik V. Liu H. Kong W. Wang Y.. (2015). Integrated, systems metabolic picture of acetone-butanol-ethanol fermentation by *Clostridium acetobutylicum*. Proc. Natl. Acad. Sci. U.S.A. 112, 8505–8510. 10.1073/pnas.1423143112 26100881 PMC4500237

[B42] Loow Y. L. Wu T. Y. Jahim J. M. Mohammad A. W. Teoh W. H. (2016). Typical conversion of lignocellulosic biomass into reducing sugars using dilute acid hydrolysis and alkaline pretreatment. Cellulose 23, 1491–1520. 10.1007/s10570-016-0936-8

[B43] Marcellin E. Behrendorff J. B. Nagaraju S. Detissera S. Segovia S. Palfreyman R. W.. (2016). Low carbon fuels and commodity chemicals from waste gases-systematic approach to understand energy metabolism in a model acetogen. Green Chem. 18, 3020–3028. 10.1039/C5GC02708J

[B44] Milne C. B. Eddy J. A. Raju R. Ardekani S. Kim P. J. Senger R. S.. (2011). Metabolic network reconstruction and genome-scale model of butanol-producing strain *Clostridium beijerinckii* NCIMB 8052. BMC Syst. Biol. 5:130. 10.1186/1752-0509-5-130 21846360 PMC3212993

[B45] Monot F. Martin J. R. Petitdemange H. Gay R. (1982). Acetone and butanol production by *Clostridium acetobutylicum* in a synthetic medium. Appl. Environ. Microbiol. 44, 1318–1324. 10.1128/aem.44.6.1318-1324.1982 16346149 PMC242190

[B46] Mook A. Beck M. H. Baker J. P. Minton N. P. Dürre P. Bengelsdorf F. R. (2022). Autotrophic lactate production from H_2_ + CO_2_ using recombinant and fluorescent FAST-tagged *Acetobacterium woodii* strains. Appl. Microbiol. Biotechnol. 10.1007/s00253-022-11770-z 35092454 PMC8882112

[B47] Moreira J. P. Diender M. Arantes A. L. Boeren S. Stams A. J. Alves M. M.. (2021). Propionate production from carbon monoxide by synthetic cocultures of *Acetobacterium wieringae* and propionigenic bacteria. Appl. Environ. Microbiol. 87:e0283920. 10.1128/AEM.02839-20 33990298 PMC8231444

[B48] Patakova P. Branska B. Sedlar K. Vasylkivska M. Jureckova K. Kolek J.. (2019). Acidogenesis, solventogenesis, metabolic stress response and life cycle changes in *Clostridium beijerinckii* NRRL B-598 at the transcriptomic level. Sci. Rep. 9, 1–21. 10.1038/s41598-018-37679-0 30718562 PMC6362236

[B49] Ragsdale S. W. Pierce E. (2008). Acetogenesis and the wood-jungdahl pathway of co2 fixation. Biochim. Biophys. Acta 1784, 1873–1898. 10.1016/j.bbapap.2008.08.012 18801467 PMC2646786

[B50] Richardson Y. Drobek M. Julbe A. Blin J. Pinta F. (2015). “Chapter 8: Biomass gasification to produce Syngas,” in Recent Advances in Thermo-Chemical Conversion of Biomass, eds A. Pandey, T. Bhaskar, M. Stãcker, and R. K. Sukumaran (Boston, MA: Elsevier), 213–250. 10.1016/B978-0-444-63289-0.00008-9

[B51] Sakarika M. Delmoitié B. Ntagia E. Chatzigiannidou I. Gabet X. Ganigué R.. (2022). Production of microbial protein from fermented grass. Chem. Eng. J. 433:133631. 10.1016/j.cej.2021.133631

[B52] Sar T. Harirchi S. Ramezani M. Bulkan G. Akbas M. Y. Pandey A.. (2022). Potential utilization of dairy industries by-products and wastes through microbial processes: a critical review. Sci. Total Environ. 810:152253. 10.1016/j.scitotenv.2021.152253 34902412

[B53] Schwalm N. D. Mojadedi W. Gerlach E. S. Benyamin M. Perisin M. A. Akingbade K. L. (2019). Developing a microbial consortium for enhanced metabolite production from simulated food waste. Fermentation 5:98. 10.3390/fermentation5040098

[B54] Show K. Y. Lee D. J. Tay J. H. Lin C. Y. Chang J. S. (2012). Biohydrogen production: current perspectives and the way forward. Int. J. Hydrog. Energy 37, 15616–15631. 10.1016/j.ijhydene.2012.04.109

[B55] Siemerink M. A. Schwarz K. Grimmler C. Kuit W. Ehrenreich A. Kengen S. W. (2014). “Chapter 8: Comparative genomic analysis of the central metabolism of the solventogenic species *Clostridium acetobutylicum* ATCC 824 and *Clostridium beijerinckii* NCIMB 8052,” in Systems Biology of Clostridium, 193–219. 10.1142/9781783264414_0008

[B56] Sreekumar S. Baer Z. C. Pazhamalai A. Gunbas G. Grippo A. Blanch H. W.. (2015). Production of an acetone-butanol-ethanol mixture from *Clostridium acetobutylicum* and its conversion to high-value biofuels. Nat. Protoc. 10, 528–537. 10.1038/nprot.2015.029 25719271

[B57] Tracy B. P. Jones S. W. Fast A. G. Indurthi D. C. Papoutsakis E. T. (2012). Clostridia: the importance of their exceptional substrate and metabolite diversity for biofuel and biorefinery applications. Curr. Opin. Biotechnol. 23, 364–381. 10.1016/j.copbio.2011.10.008 22079352

[B58] Valgepea K. Lemgruber R. D. S. P. Abdalla T. Binos S. Takemori N. Takemori A.. (2018). H2 drives metabolic rearrangements in gas-fermenting *Clostridium autoethanogenum. Biotechnol. Biofuels* 11:55. 10.1186/s13068-018-1052-9 29507607 PMC5831606

[B59] Valgepea K. Loi K. Q. Behrendorff J. B. Lemgruber R. d. S. Plan M. Hodson M. P.. (2017). Arginine deiminase pathway provides ATP and boosts growth of the gas-fermenting acetogen *Clostridium autoethanogenum*. Metab. Eng. 41, 202–211. 10.1016/j.ymben.2017.04.007 28442386

[B60] Xu Z. Luo Y. Mao Y. Peng R. Chen J. Soteyome T.. (2020). Spoilage lactic acid bacteria in the brewing industry. J. Microbiol. Biotechnol. 30, 955–961. 10.4014/jmb.1908.08069 31986245 PMC9728350

[B61] Yang Y. Hoogewind A. Moon Y. H. Day D. (2016). Production of butanol and isopropanol with an immobilized *Clostridium*. Bioprocess Biosyst. Eng. 39, 421–428. 10.1007/s00449-015-1525-1 26712323

